# Supramolecular Zinc–Polyphenol Coordination Assemblies Mitigate Colonic Inflammation by Regulating Mucosal Barrier and Microbiome

**DOI:** 10.1002/advs.77647

**Published:** 2026-09-27

**Authors:** Qiwen Xie, Mai Ye, Xuexia Liu, Jing Wang, Ying Chen, Lu Tan, Jinheng Fu, Xiaochang Huang

**Affiliations:** ^1^ State Key Laboratory of Food Science and Resources Nanchang University Nanchang China; ^2^ College of Life Sciences Sichuan University Chengdu China; ^3^ Shenzhen Hospital Southern Medical University Shenzhen Guangdong China

**Keywords:** colitis prevention, dysbiosis regulation, epithelial barrier integrity, metal–phenolic networks (MPNs), RONS scavenging, zinc coordination

## Abstract

To overcome the poor bioavailability and environmental instability of physical polyphenol co‐assemblies while maximizing their preventive efficacy, the authors engineered carrier‐free, metal–phenolic coordination nanoparticles (ZnCA NPs) utilizing divalent zinc (Zn^2+^) to bridge curcumin and anthocyanin. This coordination chemistry transforms the binary polyphenols into a stable, quasi‐amorphous state, substantially enhancing their aqueous dispersibility and gastrointestinal stability. Benefiting from this structural evolution, ZnCA NPs exhibited superior colonic accumulation and efficient reactive oxygen and nitrogen species scavenging. In zebrafish and murine colitis models, ZnCA NPs effectively reinforced the mucosal barrier and mitigated microenvironmental inflammation. Mechanistically, transcriptomic profiling revealed that ZnCA NPs modulated the colonic microenvironment by downregulating core NF‐κB, MAPK, and JAK‐STAT signaling cascades. Crucially, ZnCA NPs enriched beneficial commensals, most prominently Muribaculaceae, to attenuate dextran sulfate sodium‐induced dysbiosis; the functional contribution of this remodeled microbiota in maintaining colonic homeostasis was microbiota transplantation. This study transitions polyphenol nanomedicine from fragile physical self‐assembly to robust metal–phenolic coordination for enhanced prevention of intestinal inflammation.

## Graphical abstract

A carrier‐free zinc‐coordinated polyphenol nanoplatform (ZnCA NPs) is designed as an “all‐in‐one” therapeutic strategy for intestinal inflammation. The ZnCA NPs exhibit enhanced colonic accumulation and broad‐spectrum reactive oxygen and nitrogen species scavenging. These nanoparticles restore mucosal barrier integrity, suppress inflammatory pathways, and reshape the gut microbiota. Fecal microbiota transplantation further confirms the therapeutic efficacy of this biocompatible nanoplatform.

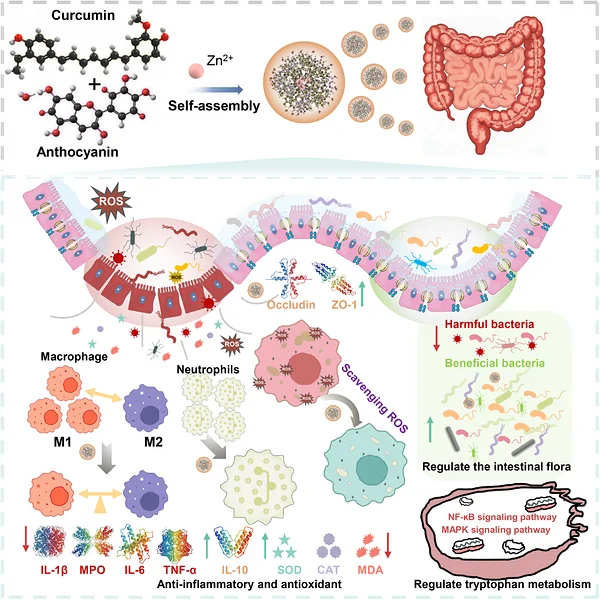

## 1 Introduction

Inflammatory bowel disease (IBD), encompassing Crohn's disease and ulcerative colitis, comprises a group of chronic, idiopathic, and relapsing intestinal disorders that severely impair patients’ quality of life and impose a substantial burden on healthcare systems worldwide [1, 2, 3, 4]. Its pathogenesis involves a complex interplay among excessive oxidative stress, gut dysbiosis, disruption of the intestinal mucosal barrier, and dysregulated local immune responses [5, 6]. Although conventional therapies—including 5‐aminosalicylic acid, corticosteroids, and biologics—primarily aim to control intestinal inflammation [5, 7, 8], their clinical utility is often limited by variable efficacy, systemic adverse effects, and incomplete mucosal healing [2, 4, 9]. Therapeutic systems capable of simultaneously restoring redox balance, repairing the epithelial barrier, and reestablishing microbial homeostasis are therefore highly desirable for the management of IBD.

Dietary polyphenols have attracted considerable interest as potential therapeutic agents for IBD because of their antioxidant and anti‐inflammatory properties and their ability to modulate the gut microbiota [10, 11, 12, 13]. Owing to their limited absorption in the small intestine, a substantial proportion of orally administered polyphenols can reach the colon, where they may protect intestinal epithelial cells from reactive oxygen species (ROS)‐mediated injury, inhibit pathogenic bacteria, and support the growth of beneficial commensals [10, 14]. These activities collectively contribute to the alleviation of intestinal inflammation and the restoration of microbial homeostasis [15]. However, the poor aqueous solubility, limited environmental stability, and low bioavailability of many polyphenols constrain their therapeutic application. Nanostructured delivery and carrier‐free self‐assembly have been explored to address these limitations, while metal–ion coordination provides an additional means of regulating the organization, stability, and biological performance of polyphenol‐based assemblies [16, 17, 18]. Nevertheless, assemblies governed primarily by noncovalent interactions may remain susceptible to dissociation under complex physiological conditions. Introducing therapeutically active metal ions as coordination nodes may therefore improve structural stability while conferring complementary biological functions.

Zinc ions (Zn^2+^) are intracellular signaling mediators involved in numerous biological processes, including the maintenance of intestinal epithelial development, function, and integrity [15]. Zinc deficiency is frequently observed in patients with IBD, with a reported prevalence of approximately 15%–45% [7]. Such deficiency can impair intestinal stem cell growth and compromise tight‐junction integrity, thereby increasing epithelial permeability [15]. Because zinc also contributes to intracellular redox homeostasis, insufficient zinc availability may aggravate oxidative stress and cellular dysfunction [5]. Accordingly, increasing evidence indicates that zinc supplementation can alleviate intestinal inflammation by restoring epithelial barrier function and regulating inflammatory responses [15, 19]. Hu et al. demonstrated that zinc protected against experimental IBD by inhibiting nuclear factor‐κB (NF‐κB) and calpain activity and increasing tight‐junction protein expression [15]. Beyond conventional supplementation, Zhang et al. developed hollow Zn–tannic acid nanoparticles with multienzyme‐like activity that continuously scavenged reactive oxygen and nitrogen species (RONS), reduced colonic injury and proinflammatory cytokine production, and promoted mucosal healing [5]. Other therapeutically active metal ions have likewise been incorporated into carrier‐free natural‐product systems. For example, Jia et al. reported a Mg^2+^‐coordinated hydrogel assembled from glycyrrhizic acid and rosmarinic acid that targeted the mitochondrial ROS–pyroptosis axis, suppressed IL‐1β secretion, and restored both the epithelial barrier and gut microbial balance [9]. Together, these studies suggest that metal coordination offers a versatile strategy for constructing multifunctional natural‐product‐based systems for intestinal inflammation.

**SCHEME 1:**
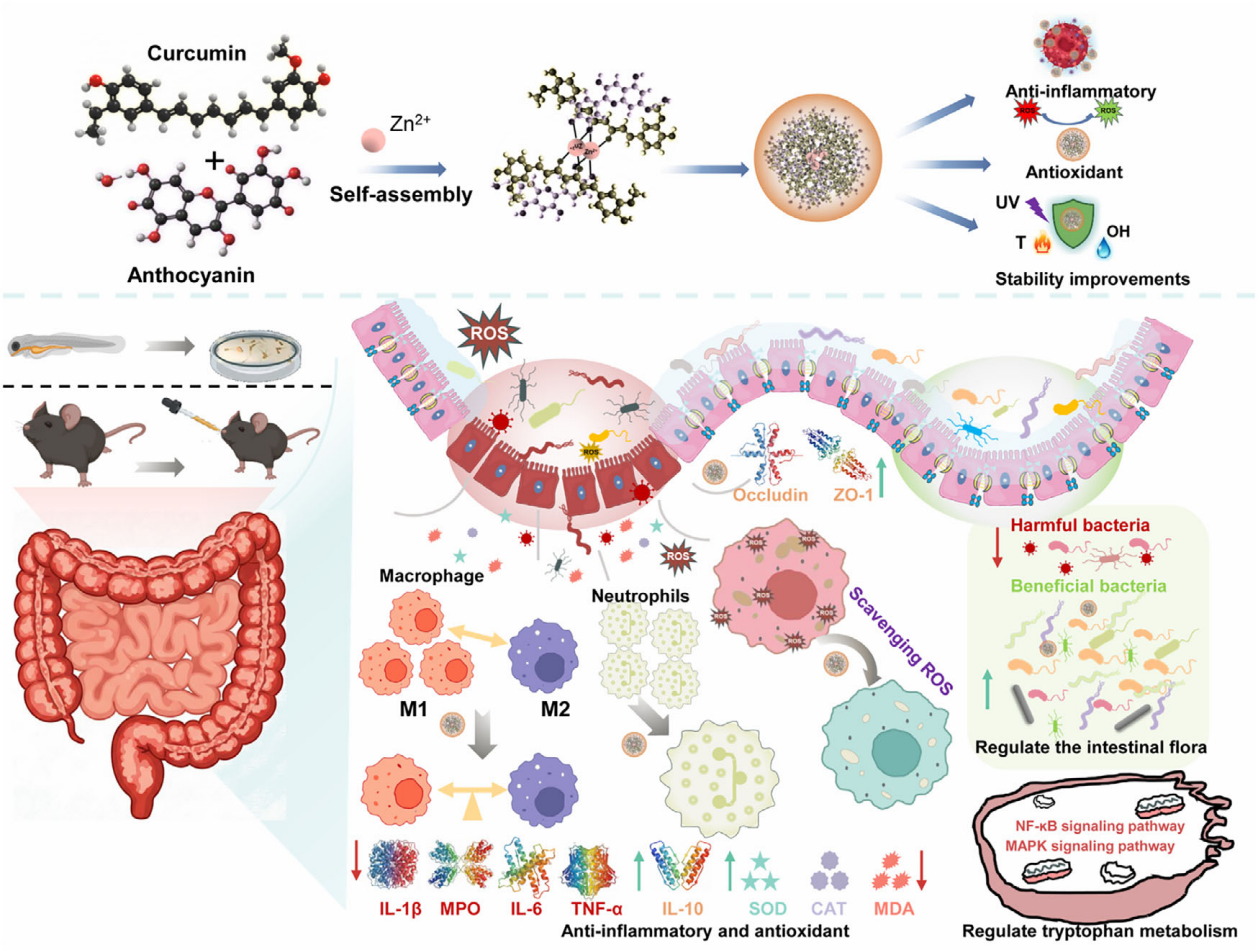
Coordination‐driven assembly of zinc‐mediated binary polyphenol nanoparticles (ZnCA) for synergistic ulcerative colitis prevention via targeted RONS scavenging, mucosal barrier reconstruction, and microbiota remodeling.

Herein, we employed Zn^2+^ as a therapeutically active coordination node to construct zinc‐coordinated curcumin–anthocyanin nanoparticles (ZnCA NPs). During assembly, the conjugated β‐diketone moieties of curcumin (Cur) and the catechol groups of anthocyanin (Ant) coordinate with Zn^2+^ to form a multiligand metal–polyphenol network [20]. This coordination‐driven reorganization, together with steric constraints among the constituent molecules, suppresses long‐range crystalline ordering and promotes the formation of a stable, quasi‐amorphous nanonetwork [16, 17]. As a result, ZnCA NPs exhibit improved aqueous dispersibility and environmental stability without the use of inert synthetic polymeric carriers [21]. The nanoparticles efficiently scavenged multiple RONS and showed enhanced accumulation in inflamed colonic tissues. At the disease site, ZnCA alleviated oxidative stress, restored epithelial tight‐junction integrity, suppressed intestinal inflammation, and remodeled the dysbiotic gut microbiota, including the recovery of beneficial members of the family Muribaculaceae (Scheme 1). Comparisons among ZnCA NPs, noncoordinated CA nanoparticles, and the Zn+CA physical mixture further distinguished the effects of Zn^2+^‐mediated coordination from the additive effects of the individual components. Moreover, fecal microbiota transplantation provided functional evidence that the microbiota remodeled by ZnCA contributed to protection against colitis. Collectively, these findings demonstrate the potential of multiligand metal–polyphenol coordination assemblies as multifunctional therapeutic systems for inflammatory intestinal disorders.

## 2 Experimental Section

### 2.1 Materials and Reagents

All chemical reagents and commercial assay kits were utilized as received from vendors without further purification. Curcumin (458‐37‐7), anthocyanin (standardized blueberry extract, indexed as cyanidin cation, CAS 13306‐05‐3, specified total anthocyanin content: 5%–25%), 1,1‐diphenyl‐2‐picrylhydrazyl (DPPH), 2,2′‐azino‐bis(3‐ethylbenzothiazoline‐6‐sulfonic acid) (ABTS), and 3,3′,5,5′‐tetramethylbenzidine were supplied by Shanghai Macklin Biochemical Technology Co., Ltd. Zinc acetate dihydrate [Zn(OAc)_2_·2H_2_O] was obtained from Sinopharm Chemical Reagent Co., Ltd., while dextran sulfate sodium (DSS, 40 kDa) was purchased from MP Biomedicals (Shanghai, China) Co., Ltd. Hydrogen peroxide (H_2_O_2_, 30%) was procured from Chengdu Jinshan Chemical Reagent Co., Ltd. Absolute ethanol, dimethyl sulfoxide (DMSO), and sodium hydroxide (NaOH, 99%) were sourced from Kelong Chemical Reagent Co., Ltd. (Chengdu, China). Regenerated cellulose dialysis membranes (molecular weight cut‐off, MWCO = 3500 Da) were purchased from Labshark (Changde Bikeman Biotechnology Co., Ltd., Changde, China). For biochemical evaluations, the following diagnostic kits were acquired from Nanjing Jiancheng Bioengineering Institute Co., Ltd.: total superoxide dismutase (T‐SOD, hydroxylamine method), catalase (CAT, visible light method), malondialdehyde (MDA, TBA method), ROS, and total protein (TP).

### 2.2 Preparation of CA and ZnCA nanoparticles

Carrier‐free curcumin–anthocyanin nanoparticles (CA NPs) were prepared according to our previously established noncovalent self‐assembly strategy [18]. Building on this platform, Zn^2+^ was introduced during the assembly process as a coordination node to construct ZnCA NPs. Briefly, curcumin (368.4 mg) and anthocyanin (861.8 mg) were co‐dissolved in a binary mixture of DMSO and ultrapure water (3:2, v/v). For the preparation of ZnCA NPs with different coordination levels, zinc acetate dihydrate was added at feeding amounts of 5.49, 10.98, 21.95, 43.90, 109.80, or 219.50 mg, corresponding to initial Zn^2^^+^ feeding concentrations of 2.5, 5, 10, 20, 50, and 100 mm, respectively. The zinc precursor was omitted when preparing the parental CA nanoparticles. Each mixture was stirred vigorously at 70 °C for 10 min, followed by continuous ultrasonication for 5 min to facilitate nanoparticle assembly. During this process, CA formation was primarily governed by noncovalent interactions, including π–π stacking and hydrophobic interactions, whereas the incorporation of Zn^2+^ introduced additional metal–phenolic coordination into the assembly network [22]. Residual solvents, unreacted components, and free Zn^2+^ were subsequently removed by dialysis against ultrapure water for 48 h using a dialysis membrane with a molecular weight cutoff of 3500 Da. The resulting CA and ZnCA nanoparticles were collected by lyophilization.

### 2.3 Characterization of NPs

Nanoparticle morphology and spatial elemental distributions were resolved through field‐emission scanning electron microscopy (FESEM, Hitachi S‐4800) equipped with an Oxford Instruments energy‐dispersive X‐ray (EDS) detector. The corresponding hydrodynamic screening and zeta potentials were determined on a Malvern Panalytical Nano ZS90 platform. UV–vis–NIR absorption profiles and emission responses were recorded using a Hitachi UH4150 spectrophotometer and an Edinburgh Instruments FLS1000 spectrofluorometer, respectively. Crystalline phases were examined by X‐ray diffraction (XRD) utilizing a Bruker D8 Advance system, whereas molecular bonds and surface compositions were monitored via Fourier transform infrared spectroscopy (FTIR, Thermo Fisher Scientific Nicolet iS50) and X‐ray photoelectron spectroscopy (XPS, Thermo Scientific ESCALAB 250Xi+). ^1^H NMR spectra were documented on a 600 MHz JEOL JNM‐ECZ600R spectrometer. Finally, thermal characteristics were mapped via thermogravimetric analysis (TGA) on a Discovery TGA55 analyzer, complemented by differential scanning calorimetry (DSC) utilizing a TA Instruments DSC250.

### 2.4 Molecular Dynamics Simulation

To elucidate the thermodynamic stability and coordination behavior of the complex, atomistic MD simulations were performed using GROMACS 2020.6. The initial configurations, encompassing Cur, Ant, and zinc acetate, were modeled within a cubic box subjected to periodic boundary conditions. The restrained electrostatic potential (RESP) method was employed to calculate atomic partial charges, while the rest of the small‐molecule topological parameters were parametrized via ACPYPE. Following energy minimization, the system was fully equilibrated through successive *NVT* and *NPT* runs. A 100 ns production run was then performed, maintaining the ambient environment at 298.15 K and 1.0 bar. Full technical details, including system setup and force‐field parameters, are provided in the Supplementary Information.

### 2.5 ABTS Radical Scavenging Assay

The total antioxidant capacity of the self‐assembled ZnCA complex was quantified via the ABTS^+^• radical cation scavenging assay. To generate the stable ABTS^+^• chromophore, equal volumes of 7.4 mm ABTS and 2.6 mm ammonium persulfate were blended and allowed to react at 4 °C in the dark for 12–16 h. Prior to the scavenging assessment, this radical stock solution was equilibrated with phosphate‐buffered saline (PBS) to anchor its optical density (OD) at 0.70 ± 0.05 at a wavelength of 734 nm. For the anti‐radical evaluation, 100 µL aliquots of aqueous ZnCA suspensions were introduced into a 96‐well plate, followed by the addition of 100 µL of the prepared ABTS working solution, establishing a final reaction concentration gradient of 0, 10, 20, 40, and 80 µg/mL. After a 10 min incubation at 25 °C in the absence of light, the residual absorbance at 734 nm was monitored using a microplate spectrophotometer.

### 2.6 DPPH Free Radical Scavenging Activity

To quantify the anti‐radical performance of ZnCA, a modified DPPH assay was executed in a 96‐well plate. Aliquots of aqueous ZnCA (100 µL) were reacted with 100 µL of an ethanolic DPPH radical stock solution (200 µg/mL) to yield designated final working concentrations ranging from 0 to 100 µg/mL. Following a 15‐min incubation period maintained at 25 °C in the absence of light, the remaining concentration of stable DPPH radicals was tracked by monitoring the optical attenuation at 517 nm using a microplate spectrophotometer.

### 2.7 Hydroxyl Radical (·OH) Generation Assay

The ·OH radical scavenging kinetics of ZnCA were determined via an aqueous sodium salicylate trapping assay. To rule out solvent‐induced competitive inhibition, the radical probe was dissolved strictly in water (50 mm). ZnCA variants (1.25–10 µg/mL) were freshly harvested from a water‐diluted parent stock (100 µg/mL, initially wetted in 1 mL ethanol). The assay was conducted by assembling 200 µL of sample, 80 µL of the probe, and 520 µL of the Fenton pairs (FeSO_4_·7H_2_O and H_2_O_2_) within 1.5 mL tubes, instantly inducing ·OH evolution. Crucially, the reaction environment was regulated at pH 3.0 using trace HCl to maximize Fenton catalytic activity. After reacting at 25 °C for 10 min in the dark, the solutions were transitioned to a multi‐well platform, and the optical attenuation at 510 nm was read on a microplate reader against ZnCA blanks.

### 2.8 Nitric Oxide Radical (NO•) Scavenging Activity

To evaluate the nitric oxide (NO•) radical scavenging efficiency of the self‐assembled ZnCA NPs, a modified Griess assay adapted from the protocol described by Wu et al. [7] was performed. Briefly, NO• radicals were generated using a sodium nitroprusside (SNP) donor system. In a typical procedure, 100 µL of the SNP donor solution (10 mm in PBS, pH 7.4) was thoroughly mixed with an equal volume of ZnCA nanoparticles (to achieve a final concentration of 12.5 µg/mL) and incubated at room temperature for 2 h under continuous light illumination to facilitate NO• generation. Subsequently, the diazotization reaction was initiated by adding 100 µL of freshly prepared Griess reagent (a 1:1 mixture of Reagent A [10 mg/mL sulfanilic acid in 5% H_3_PO_4_] and Reagent B [1 mg/mL N‐1‐naphthylethylenediamine dihydrochloride]). Following a 15 min incubation period in the dark to allow for complete chromogenic development, the absorbance at 540 nm was spectrophotometrically recorded using a microplate reader.

### 2.9 In Vivo Efficacy against Intestinal Inflammation in a Zebrafish Model

#### 2.9.1 Zebrafish Husbandry

This study involved the use of wild‐type (AB) and transgenic *Tg*(*lyz:DsRed2*) zebrafish (*Danio rerio*) obtained from the National Zebrafish Resource Center. Animals were housed and sustained under a regulated 14:10 h light/dark cycle (ZT0 at light‐on, ZT14 at light‐off) at a constant temperature of 28 ± 1°C. To obtain embryos, sexually mature adults were paired overnight in mating chambers (at a male‐to‐female ratio of 1:1 or 1:2) using a partition board. Spawning was initiated by removing the isolation boards at the onset of the light cycle the next day, after which the resulting embryos were collected [23]. To prevent pigment differentiation for downstream analysis, the developing larvae were maintained in embryo medium containing 0.2 mm 1‐phenyl‐2‐thiourea (PTU). All animal husbandry practices and experimental manipulations strictly adhered to the ethical frameworks authorized by the Laboratory Animal Management Committee of Nanchang University.

#### 2.9.2 Zebrafish Embryo Toxicity Test

The in vivo biocompatibility of CA and ZnCA NPs was evaluated through acute toxicity and developmental assays using zebrafish (*Danio rerio*) [23, 24, 25]. For the larval assay, survival was recorded over a 96 h exposure period beginning at 3 days post‐fertilization (dpf). Concurrently, embryonic development and hatching success were tracked following exposure initiated at 2 h post‐fertilization (hpf). Both assays tested a series of concentrations ranging from 50 to 1000 µg/L. To prevent solvent‐induced artifacts, the final concentration of DMSO was strictly maintained below 0.1% (v/v) across all experimental groups. Additional experimental parameters, including medium composition and specific incubation regimes, are detailed in the Supplementary Information.

#### 2.9.3 Establishment of a Zebrafish Model of IBD

At 3 dpf, wild‐type AB strain or transgenic *Tg*(*lyz:DsRed2*) zebrafish larvae were randomly assigned into six parallel groups (*n* = 30 per group) in six‐well plates: (1) control (untreated culture medium); (2) model [0.4% (w/v) dextran sulfate sodium, DSS]; (3) CA NPs (0.4% DSS with 350 µg/L CA NPs); (4) physical mixture of Zn^2+^ and CA (Zn+CA) [0.4% DSS with a physical mixture of 350 µg/L CA NPs and free Zn^2+^]; (5) ZnCA (L) (0.4% DSS with 350 µg/L ZnCA NPs); and (6) ZnCA (H) (0.4% DSS with 700 µg/L ZnCA NPs). To ensure a rigorous control, the concentration of free Zn^2+^ in the Zn+CA group was precisely formulated to match the elemental zinc content in the ZnCA (L) group. The larvae underwent continuous waterborne exposure to these designated formulations, with the incubation medium completely refreshed every 24 h to maintain a constant effective concentration. Following a 5‐day exposure regimen, the larvae were harvested at 8 dpf for subsequent phenotypic and physiological evaluations.

### 2.10 In Vivo ROS Fluorescence Imaging

As previously described [23, 24, 26], the generation of ROS in live zebrafish was visualized using the fluorogenic probe 2′,7′‐dichlorodihydrofluorescein diacetate (DCFH‐DA) (E004‐1‐1, Nanjing Jiancheng Bioengineering Institute). Live larvae were harvested and exposed to 10 µm DCFH‐DA for 30 min under dark conditions at a constant temperature of 28 °C. After washing three times with the culture solution to remove excess probe, the specimens were immobilized using 0.016% Tricaine anesthetic. Morphological and fluorescent imaging was performed on a ZEISS AXIO Zoom.V16 fluorescence stereomicroscope operating at excitation and emission wavelengths of 488 nm and 525 nm, respectively. Based on the acquired micrographs, the total fluorescence intensity (TFI) specifically within the intestinal region was calculated utilizing ImageJ software (NIH, Bethesda, MD, USA). Each group comprised ten individual larvae, and data were obtained from three independent experimental runs.

### 2.11 Migration of Intestinal Immune Cells

Transgenic *Tg*(*lyz:DsRed2*) zebrafish embryos, which specifically exhibit red fluorescence in neutrophils, were obtained via natural spawning according to standard laboratory breeding protocols. Following the designated administration, 8 dpf larvae from all experimental groups were anesthetized by immersion in 0.016% (w/v) Tricaine. To facilitate oriented fluorescent imaging, the anesthetized larvae were gently embedded in 1% low‐melting‐point agarose and promptly imaged using a ZEISS AXIO Zoom.V16 stereo fluorescence microscope. For each treatment group, 15 individual larvae were randomly selected, and all assays were independently performed in triplicate (*n* = 15 per group, three biological replicates). The total number of DsRed‐positive neutrophils infiltrating the larval intestinal tract was precisely quantified using Image‐Pro Plus 6.0 software (Media Cybernetics, USA).

### 2.12 Quantitative Real‐Time Polymerase Chain Reaction

For each experimental group, three biological replicates were processed, each containing a pool of 30 zebrafish larvae in a 2 mL centrifuge tube. Total RNA was isolated using the TRIzol reagent (Invitrogen, USA) and subsequently reverse‐transcribed into cDNA using the HiScript II Q RT SuperMix for qPCR kit (Vazyme, China). Quantitative real‐time PCR (qPCR) was performed using the SYBR PCR master kit (Thermo Fisher, USA). The specific primer sequences (5′‐3′) are provided in Table S1. Relative mRNA expression levels were quantified using the comparative C_T_ (2^−△△CT^) method, with β‐actin serving as the internal reference gene. Data are expressed as fold changes relative to the control group [24].

### 2.13 Oxidative Stress Analysis

To evaluate oxidative stress levels, the activities of superoxide dismutase (SOD; Cat. No. A001‐3‐2) and CAT (Cat. No. A007‐1‐1), alongside the content of MDA (Cat. No. A003‐1‐2), were determined in zebrafish larvae. All assays were performed using commercial kits purchased from the Nanjing Jiancheng Bioengineering Institute (Nanjing, China) following the manufacturer's instructions. For each of the three independent experiments, a pool of 50 zebrafish larvae per group was homogenized. Absorbance was quantified using a SpectraMax iD3 multi‐mode microplate reader (Molecular Devices, USA), and the data were normalized to the TP content of each sample [23, 26].

### 2.14 Animal Study on Mice

Male C57BL/6 mice (7–8 weeks old, weighing 22 ± 2 g) were purchased from GemPharmatech Co., Ltd. (Nanjing, China). Prior to experimental initiation, the animals were acclimated for 7 days in a standardized facility under controlled environmental conditions (temperature: 25 ± 1 °C; relative humidity: 40%–70%; 12‐h light/dark cycle). The mice were allowed ad libitum access to standard laboratory chow and water. All animal procedures were conducted in strict accordance with the National Regulation of China for the Care and Use of Laboratory Animals. The experimental protocols were reviewed and formally approved by the Animal Ethics Committee of Nanchang University (Approval No. HC2572/‐P07/‐0).

### 2.15 DSS‐Induced IBD Mouse Model Establishment

To evaluate the beneficial outcomes, 64 male C57BL/6 mice were randomly assigned to eight experimental groups (*n* = 8 per group): (1) Control (PBS vehicle), (2) DSS (Model), (3) Free Zn (Zn dosage matched to ZnCA (M)), (4) CA (10 mg/kg), (5) Zn+CA (physical mixture, matched to ZnCA (M)), (6) ZnCA (Low dose, 5 mg/kg), (7) ZnCA (Medium dose, 10 mg/kg), and (8) ZnCA (High dose, 20 mg/kg). The experimental regimen comprised a 3‐day prophylactic pre‐treatment phase followed by a 7‐day concurrent treatment and DSS‐induction phase. From Day 1 to Day 3, mice in the intervention groups (Groups 3–8) were administered their designated formulations daily via oral gavage, while the Control and DSS groups received an equivalent volume of PBS (0.01 m, pH 7.4). On Day 4, acute colitis was induced in all groups except the Control group by supplementing the drinking water with 3.5% (w/v) dextran sulfate sodium (DSS, MW ∼40 kDa) for 7 consecutive days, during which the daily gavage interventions were sustained [27]. The disease activity index (DAI) was assessed daily from Day 1 to Day 10 based on body weight loss, stool consistency, and macroscopically visible blood in the stool, according to the scoring criteria provided in Table S2 [28, 29]. On Day 11, all mice were euthanized. The colons were excised and measured for length, while the heart, liver, spleen, lung, and kidney were collected and weighed to determine the organ index as follows [22]:

$$Organindex=organweight\left(g\right)/bodyweight\left(g\right)\times 100\%$$

### 2.16 Enzyme‐Linked Immunosorbent Assay (ELISA) Test of Cytokines

To evaluate the local inflammatory profile, the concentrations of interleukin‐1β (IL‐1β), IL‐6, IL‐10, and tumor necrosis factor‐alpha (TNF‐α) in colon tissue homogenates were measured. Assays were performed using commercial ELISA kits (Servicebio Biotech, Wuhan, China) in strict accordance with the provided protocols. Optical density was captured at 450 nm via a Tecan Infinite M200 Pro microplate reader, and cytokine abundance was determined based on concurrent standard curves [22].

### 2.17 Histological and Histochemical Analysis

To characterize mucosal architecture and barrier function, colonic sections (4 µm) from paraffin blocks were subjected to hematoxylin and eosin (H&E) and Alcian Blue‐Periodic Acid Schiff (AB‐PAS) staining. While H&E sections served to evaluate generalized tissue damage and leukocyte infiltration, AB‐PAS staining quantified goblet cell density and local mucin secretion. Slides were digitized using a slide‐scanning system. Assessment of histological damage was performed semi‐quantitatively using a standardized scoring system (Table S3) [28], with investigators kept blinded to the treatment allocations. Methodological specifics for tissue fixation, embedding, and scoring parameters are detailed in the Supplementary Information.

### 2.18 Immunofluorescence

The architectural integrity and spatial organization of the colonic mucosal barrier were evaluated via immunofluorescence staining on paraffin‐embedded sections. Specifically, the expression and localization patterns of the tight junction (TJ) components Occludin and zonula occludens‐1 (ZO‐1) were probed using target‐specific primary antibodies, followed by signal detection via an Alexa Fluor 488‐conjugated secondary antibody. Nuclei were resolved using 4′,6‐diamidino‐2‐phenylindole (DAPI) counterstaining. Fluorescence micrographs were captured using a laser scanning confocal microscope (LSM 900, Zeiss, Germany) to delineate protein distribution along the epithelial border. Comprehensive protocols for epitope retrieval, blocking conditions, and antibody titers are provided in the Supplementary Information.

### 2.19 ROS Measurement

To evaluate colonic ROS levels, freshly harvested tissue specimens were immediately encapsulated in optimal cutting temperature (OCT) compound and flash‐frozen in liquid nitrogen. Cryosections with a thickness of 20 µm were prepared at −20 °C utilizing a Leica cryostat. For ROS fluorometric tracing, the fresh sections were incubated with 5 µm dihydroethidium (DHE; Beyotime Biotechnology, Shanghai, China) for 30 min at 37 °C within a darkened, humidified chamber. Nuclei were counterstained with DAPI. Confocal micrographs were subsequently captured on an Olympus laser scanning confocal microscope (Tokyo, Japan) to resolve the fluorescence profiles.

### 2.20 Immunohistochemistry

To evaluate leukocyte infiltration and epithelial turnover, immunohistochemical (IHC) analysis was performed on paraffin‐embedded colonic sections. Neutrophil infiltration was assessed using a primary antibody against myeloperoxidase (MPO). Bound antibodies were visualized via a 3,3′‐diaminobenzidine (DAB) chromogenic detection system, with hematoxylin applied to resolve nuclear morphology. Representative brightfield images were captured using an optical microscope for downstream semi‐quantitative analysis. Methodological specificities including antigen retrieval parameters, antibody titers, and full staining sequences are detailed in the Supplementary Information.

### 2.21 RNA Extraction, Library Construction, and Sequencing

To explore the molecular mechanisms driving the protective efficacy of the nanoplatforms, total RNA isolated from colonic tissues was subjected to high‐throughput transcriptomic sequencing using the Illumina NovaSeq 6000 platform. Following quality filtering, clean reads were aligned to the *Mus musculus* reference genome (GRCm39), with transcript abundance quantified as transcripts per million (TPM). Differentially expressed genes (DEGs) were identified using the DESeq2 package, based on statistically stringent thresholds |log2 Fold Change| ≥ 1 and adjusted *P* < 0.05. Functional annotation and pathway elucidation were subsequently achieved through Gene Ontology (GO) and Kyoto Encyclopedia of Genes and Genomes (KEGG) enrichment analyses [28]. Detailed methodology regarding library preparation, quality control parameters, and the bioinformatic analyses is provided in the Supplementary Information.

## 3 16S rRNA Sequencing and Analysis of Mouse Fecal Samples

To investigate the modulation of the gut microbial community by ZnCA NPs, 16S rRNA gene amplicon sequencing of the V3‐V4 hypervariable regions was performed on an Illumina NovaSeq 6000 platform. Quality filtering, sequence denoising, and the generation of amplicon sequence variants (ASVs) were performed using the q2‐deblur plugin within the QIIME 2 pipeline (version 2024.2). Taxonomic classification was subsequently performed against the Greengenes database (release 13_8) using the pre‐trained gg‐13‐8‐99‐515‐806‐nb‐classifier.qza classifier. Variations in microbial community structure were evaluated through alpha diversity indices and visualized via principal coordinate analysis (PCoA) based on Bray–Curtis distances. Distinct taxonomic biomarkers exhibiting significant differential abundance across groups were identified using ANCOMBC (version 2.8.1). Furthermore, Spearman's rank correlation analysis was utilized to establish potential relationships between the microbial profiles, host clinical parameters, and inflammatory markers. Full experimental procedures regarding DNA extraction, PCR amplification conditions, and specific bioinformatic thresholds are detailed in the Supplementary Information.

### 3.1 In Vivo Biodistribution and Colonic Retention Study

To investigate the gastrointestinal transit and colonic retention of the nanoparticles, near‐infrared fluorescence tracking was performed using DiR encapsulated within CA and ZnCA NPs. Healthy and DSS‐induced acute colitis mice received free DiR or DiR‐labeled nanoparticles by oral gavage. Whole‐body fluorescence imaging was performed longitudinally for 24 h after administration using an IVIS Spectrum system. Subsequently, the excised gastrointestinal tracts were imaged ex vivo under identical imaging settings. Relative fluorescence intensities in the colonic regions were semi‐quantified using Image‐Pro Plus 6.0 software (Media Cybernetics, USA) by defining consistent regions of interest (ROIs). Detailed procedures for nanoparticle labeling and optical imaging are provided in the Supplementary Information.

### 3.2 Fecal Microbiota Transplantation

Donor fecal pellets were harvested from the ZnCA (H) group following a strict washout phase to minimize residual nanoparticle carryover, and subsequently processed into standardized bacterial slurries [30]. Concurrently, recipient C57BL/6 mice were cleared of their native gut microbiota using an intensive broad‐spectrum antibiotic regimen (ABX). Following the establishment of antibiotic‐treated, microbiota‐depleted recipient mice and a two‐day antibiotic washout period, the recipients were challenged with DSS to induce acute colitis while simultaneously receiving daily oral gavage of either the donor fecal suspension or sterile PBS as the vehicle control. The engraftment efficacy and beneficial outcomes were comprehensively evaluated through clinical disease scoring, colonic histopathological examination, mucosal barrier integrity validation, and inflammatory cytokine profiling. Comprehensive operational parameters for antibiotic administration, slurry preparation, and downstream biochemical quantification are documented in the Supplementary Information.

### 3.3 Biosafety Assessment

To evaluate the hemocompatibility profile of the self‐assembled ZnCA NPs, an in vitro hemolysis assay was performed using fresh mouse whole blood. Erythrocytes (RBCs) were isolated via centrifugation at 1000×*g* for 10 min and subsequently purified through three consecutive washing cycles with sterile 0.9% NaCl physiological saline. The resulting packed RBCs were resuspended in saline to yield a standardized 2% v/v stock suspension. For the biocompatibility evaluation, a 0.5 mL aliquot of this RBC suspension was thoroughly blended with an equal volume of saline containing ZnCA NPs at gradient concentrations. Baseline zero‐lysis and maximum‐lysis references were established by incubating identical volumes of the RBC suspension with 0.5 mL of pure saline and deionized water, respectively. The reaction matrix was maintained at 37 °C for 2 h in a water bath. Following incubation, the mixture was centrifuged to sediment intact cellular elements, and the hemoglobin release in the supernatant was monitored by quantifying the optical density at 541 nm using a UV–visible spectrophotometer. The hemolysis percentage was calculated according to the standard expression [31]:

$$\begin{array}{ccc} Hemolysis\left(\%\right) & = & \left(OD_{sample}-OD_{negative}\right)\\ & & /\left(OD_{positive}-OD_{negative}\right)\times 100\% \end{array}$$

Systemic biosafety was evaluated using blood and major organs collected from mice at the endpoint of the DSS‐induced colitis experiment. Serum samples from the Control, DSS, and ZnCA(H) groups were analyzed for hepatic function‐related parameters, including alanine aminotransferase (ALT), aspartate aminotransferase (AST), the total protein‐to‐albumin ratio (TP/ALB), and total bilirubin (T‐BIL), as well as renal function‐related parameters, including creatinine (CREA) and urea [32]. The heart, liver, spleen, lungs, and kidneys were harvested from mice in the Control, DSS, Zn, CA, Zn+CA, ZnCA(M), and ZnCA(H) groups, fixed in 4% paraformaldehyde, embedded in paraffin, sectioned, and stained with H&E. Histopathological examination was performed to identify potential treatment‐associated tissue abnormalities [33].

### 3.4 Statistical Analysis

Quantitative datasets were obtained from at least three independent biological replicates and are presented as the mean ± standard deviation (SD). To fulfill the underlying assumptions for parametric analyses, the normality of data distribution and homoscedasticity of variances were formally verified utilizing the Shapiro–Wilk test and Levene's test, respectively. For pairwise comparisons between two distinct groups, a two‐tailed unpaired Student's *t*‐test was implemented. For multi‐group evaluations (≥3), statistical variances were assessed via a one‐way analysis of variance (ANOVA) followed by Tukey's post hoc test for multiple comparisons. Statistical significance was designated at a threshold of *p* < 0.05. Graphical indicators for probability thresholds are defined as *p* < 0.05 (*), *p* < 0.01 (**), and *p* < 0.001 (***) relative to the designated control group. All analyses were performed in IBM SPSS Statistics software (Version 27.0; IBM Corp., Armonk, NY, USA). The exact sample size (*n*) and specific statistical metrics for each individual assay are explicitly delineated within the corresponding figure legends.

## 4 Results and Discussion

### 4.1 Fabrication and Characterization of ZnCA NPs

ZnCA NPs were constructed through a facile, coordination‐driven self‐assembly process (Scheme 1). Building on our previously established carrier‐free Cur–Ant nanoparticles (CA NPs), in which Cur and Ant co‐assembled primarily through noncovalent interactions, including π–π stacking and hydrogen bonding [18], we introduced Zn^2+^ as a coordination node to reorganize the supramolecular assembly network. The strong chelating affinity of the β‐diketone moiety of Cur and the catechol groups of Ant toward Zn^2+^ promoted the formation of a multiligand metal–phenolic coordination network. The immediate color change following Zn^2+^ addition provided preliminary visual evidence of changes in the coordination environment.

**FIGURE 1:**
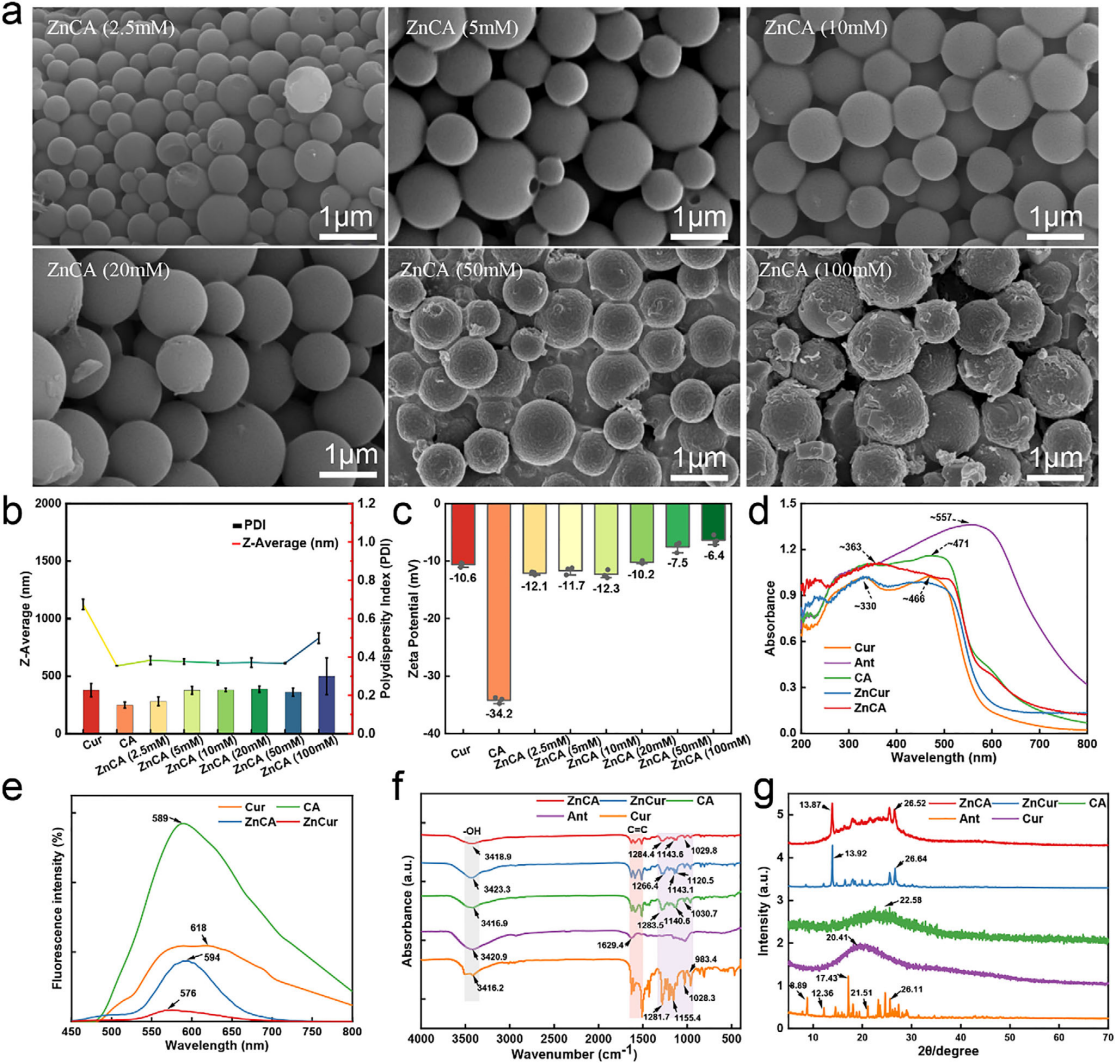
Synthesis and physicochemical characterization of zinc‐coordinated binary polyphenol nanoparticles. (a) Representative scanning electron microscopy (SEM) images of ZnCA nanoparticles synthesized with varying Zn^2+^ concentrations (2.5, 5, 10, 20, 50, and 100 mm). (b) Z‐average particle size and polydispersity index (PDI) of free Cur, CA, and ZnCA NPs prepared with varying Zn^2+^ concentrations. (c) Zeta potential of free Cur, CA, and ZnCA NPs prepared with varying Zn^2+^ concentrations. (d) UV–vis absorption and (e) fluorescence emission spectra of free Cur, Ant, CA, ZnCur, and ZnCA NPs (excluding Ant in (e)), illustrating the electronic coordination between Zn^2+^ and polyphenol ligands. (f) Fourier‐transform infrared (FTIR) spectra and (g) X‐ray diffraction (XRD) patterns of Cur, Ant, CA, ZnCur, and ZnCA NPs, highlighting the chemical bonding and amorphous/crystalline transformations during the coordination‐driven self‐assembly. Data in (b) and (c) are presented as mean ± SD (*n* = 3). Scale bars are indicated in the respective SEM panels.

Owing to its hydrophobicity, free Cur formed large aggregates in water with a Z‐average size of ∼1123.4 nm (Figure 1b). Assembly with Ant into binary CA nanoparticles substantially alleviated this aggregation, reducing the size to 589.2 nm with a minimized polydispersity index. For the ternary ZnCA system, SEM revealed a persistent spherical morphology across all Zn^2+^ feeds (2.5, 5, 10, 20, 50, and 100 mm) (Figure 1a). Within 2.5–50 mm Zn^2+^, the nanoparticles maintained relatively stable particle sizes and low PDI values. Conversely, 100 mm Zn^2+^ led to a significant increase in both size and PDI (Figure 1b), accompanied by particle adhesion in SEM, indicating that excessive coordination disrupts controlled assembly and drives secondary aggregation.

The zeta‐potential evolution (Figure 1c) reflected the surface charge transitions during sequential assembly and metal coordination. Free Cur exhibited a zeta potential of −10.6 mV, which drastically decreased to −34.2 mV upon assembling into the carrier‐free CA complex, confirming the formation of a highly stable and well‐dispersed colloid. Following Zn^2+^ introduction (2.5–10 mm), the zeta potential stabilized within a narrow range of −11.7 to −12.3 mV due to the progressive neutralization of phenolic hydroxyls by zinc cations. However, at excessive Zn^2+^ levels (20–100 mm), the surface charge was further neutralized to −6.4 mV; within this high‐concentration range, particle size increased and homogeneity declined (Figure 1b), likely from severe charge screening or excessive interparticle cross‐linking [34]. Balancing colloidal stability, optimal size, and monodispersity, the 10 mm formulation was selected as the ZnCA architecture for all subsequent biological evaluation. Furthermore, the exact chemical composition and individual encapsulation efficiencies of the components within this optimized formulation were quantitatively determined (Table S4).

In the UV–vis absorption spectra (Figure 1d), free Cur and free Ant exhibited maximum absorption peaks at ∼466 and ∼557 nm, respectively. In CA, the maximum absorption peak shifted to 471 nm, indicating that the co‐assembly altered the chromophore microenvironment [35]. Upon the addition of Zn^2+^, ZnCur displayed a distinctive coordination peak at ∼330 nm along with a broad absorption band around ∼460 nm. Similarly, ZnCA exhibited a maximum absorption peak at ∼363 nm and a wide absorption band spanning 450–470 nm. The emergence of these characteristic peaks and broad bands is typical of metal–phenolic complexes, consistent with the chelation of Zn^2+^ by the β‐diketone of Cur and the catechol of Ant, which induces extensive electronic delocalization across the network. Fluorescence spectra (Figure 1e) showed that free Cur emitted at 618 nm; CA exhibited a blueshifted, markedly intensified emission at 589 nm—an aggregation‐induced enhancement ascribed to the restriction of intramolecular rotation that suppresses nonradiative decay [36, 37]. ZnCur emission blueshifted to 576 nm and was quenched relative to free Cur, whereas ZnCA emission redshifted to 594 nm (∼5 nm vs CA) with a substantial intensity drop. The red‐shift and quenching together support the formation of a genuine ZnCA coordination entity rather than physical encapsulation, with Zn^2^^+^ bridging the ligands and modifying their excited‐state energies [38].

In the FTIR spectra (Figure 1f), the phenolic –OH stretch of crystalline Cur appeared at 3416.2 cm^−^^1^ and shifted or broadened into a prominent band in Ant (3420.9 cm^−1^), CA (3416.9 cm^−1^), ZnCur (3423.3 cm^−1^), and ZnCA (3418.9 cm^−1^), consistent with disruption of Cur's hydrogen‐bonding network and/or incorporation of coordination water [39, 40]. In the skeletal region, the Cur C–O band at 1281.7 cm^−1^ shifted to 1283.5 cm^−1^ in CA, 1266.4 cm^−1^ in ZnCur, and 1284.4 cm^−1^ in ZnCA, while the 1155.4 cm^−1^ band shifted to 1140.6 cm^−1^ (CA), 1143.1 cm^−1^ (ZnCur), and 1143.6 cm^−1^ (ZnCA), indicating an altered electronic environment around the ether/hydroxyl groups. The 983.4 cm^−^^1^ band of Cur (C–H out‐of‐plane bend of the heptadienone chain) vanished in CA but persisted in ZnCur and ZnCA, suggesting that Zn^2+^‐mediated coordination or assembly sterically constrains the Cur backbone or directly engages it in the coordination network.

In the XRD patterns (Figure 1g), crystalline Cur gave sharp reflections at 2θ = 8.89°, 12.36°, 17.43°, 21.51°, and 26.11°, whereas Ant was amorphous (broad hump at 20.41°). In CA, the sharp Cur peaks disappeared and were replaced by a broad hump at 22.58°, showing that π–π interaction with Ant directed an amorphous co‐assembly. ZnCur was crystalline with new peaks at 13.92° and 26.64°, indicating a defined zinc–curcumin complex; in ZnCA the dominant 13.87° peak (shifted from ZnCur) was strongly attenuated, with a minor peak at 26.52°. Thus Zn^2+^ orders with Cur alone, but adding Ant disrupts the lattice symmetry to give a quasi‐amorphous ZnCA whose reduced crystallinity is expected to improve aqueous dispersibility and bioactive release.

**FIGURE 2:**
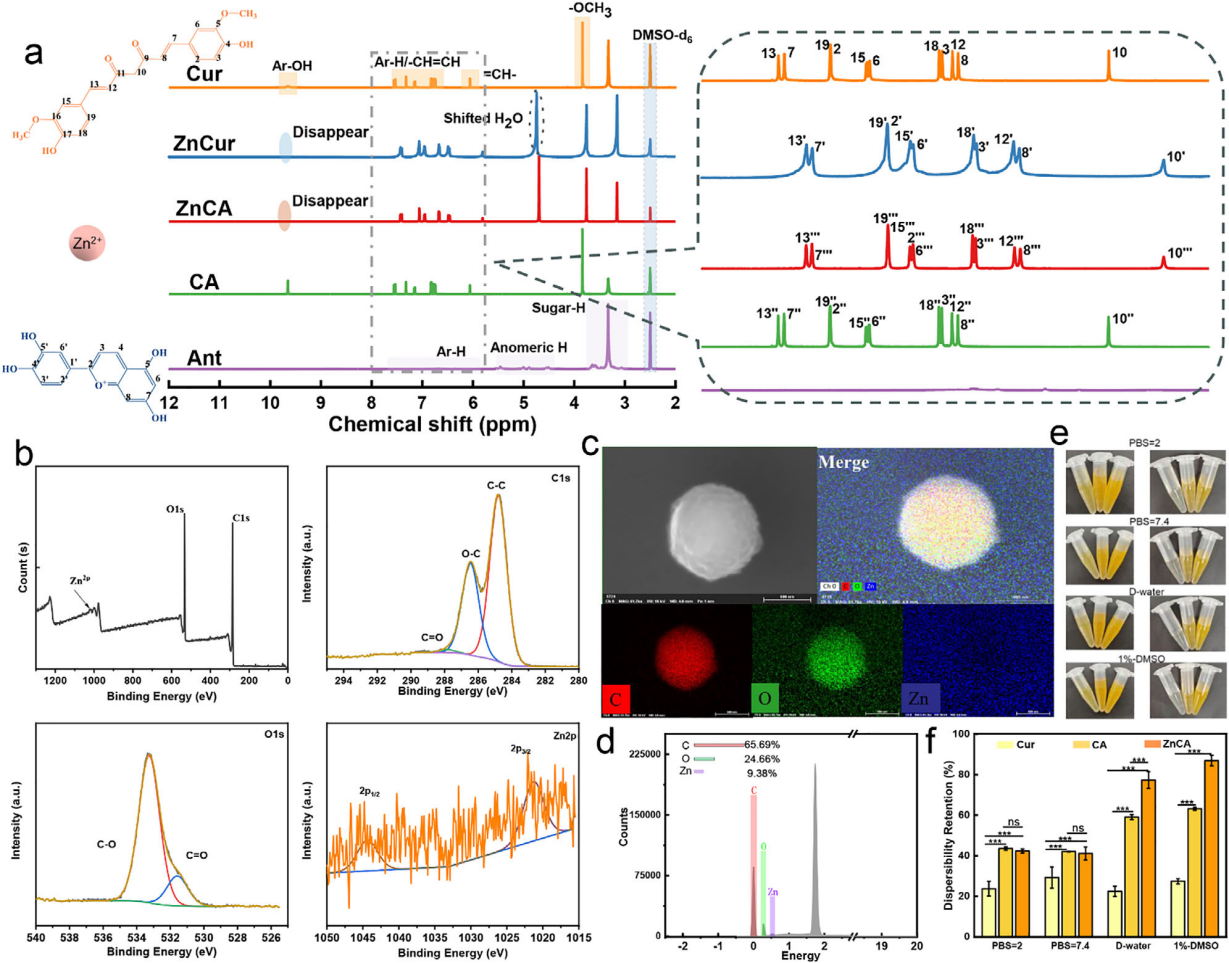
Structural validation, elemental composition, and aqueous dispersibility of ZnCA nanoparticles. (a) ^1^H nuclear magnetic resonance (^1^H NMR) spectra of free Cur, Ant, binary CA, and Zn‐coordinated formulations (ZnCur and ZnCA), showing changes in the chemical environments of characteristic protons following the introduction of Zn; the dashed box on the right shows the enlarged spectra in the aromatic proton region (4.5–7.5 ppm). (b) X‐ray photoelectron spectroscopy (XPS) survey spectrum and high‐resolution C 1s, O 1s, and Zn 2p spectra of ZnCA, supporting the successful incorporation of Zn into the nanoparticles. (c) Representative SEM image and corresponding elemental maps of C, O, and Zn in ZnCA (scale bar: 600 nm). (d) Energy‐dispersive X‐ray spectroscopy (EDS) spectrum and quantitative elemental analysis of ZnCA. (e) Visual photographs (0 h vs 2 h) and (f) quantitative analysis of the aqueous dispersibility of free Cur, binary CA, and coordinated ZnCA nanoparticles in various media after 2 h of undisturbed sedimentation, demonstrating the significantly enhanced colloidal stability and hydrophilicity achieved via coordination‐driven assembly. Data in (f) are presented as mean ± SD (*n* = 3). Statistical significance is defined as ***p < 0.001, and ns represents no significant difference.

^1^H NMR (Table S5, Figure 2a) revealed that the phenolic –OH signal of free Cur (9.64 ppm) disappeared in ZnCur and ZnCA, signifying deprotonation and Zn–O bond formation. In ZnCur, protons across the curcumin scaffold shifted upfield—enolic H‐10 from 6.06 to ≈5.81 ppm (Δδ = −0.25), H‐13/H‐7 from ≈7.56/7.52 to ≈7.42/7.40 ppm, H‐19/H‐2 from ≈7.32 to 7.06 ppm (Δδ = −0.26) and H‐15/H‐6 from ≈7.15 to 6.95 ppm (Δδ = −0.20)—consistent with zinc chelation rigidifying the heptadienone backbone. Comparing the binary and ternary systems exposed distinct assembly modes. In CA, the lack of significant chemical shift perturbations (Δδ ≈ 0.00 ppm; H‐10″ at 6.06 ppm and H‐13″/H‐7″ at 7.56/7.53 ppm) indicates negligible structural alteration of the curcumin backbone in the binary mixture. Introducing Zn^2^^+^ drove pronounced coordination‐induced shifts in the ternary system: in ZnCA, H‐10‴ resonated at ≈5.81 ppm (Δδ = −0.25) and H‐13‴/H‐7‴ at ≈7.43/7.41 ppm, demonstrating that metal–ligand coordination dominates the ternary structure rather than simple binary blending (further reflected by H‐18‴/H‐3‴ at ≈6.66 ppm and H‐12‴/H‐8‴ at 6.48/6.45 ppm).

The 2.5–5.0 ppm region was most diagnostic. Free Cur shows the primary methoxy signals (3.83 and 3.32 ppm), whereas ZnCur displays distinct shifts at 3.75 and 3.15 ppm (shielded –OCH_3_, Δδ = −0.08 and −0.17) alongside a prominent new peak at 4.74 ppm corresponding to shifted H_2_O, reflecting coordination‐induced symmetry breaking, an altered water microenvironment, and conformational locking of the curcumin backbone. These signatures persisted in ZnCA (≈3.15, 3.75, 4.69 ppm) at altered intensities. Characteristic Ant anomeric protons (≈5.2–5.5 ppm) and sugar protons (≈3.4 ppm) were lost or severely broadened in ZnCA, indicating that Ant is no longer merely mixed but tightly entrapped or coordinated within a dense zinc–polyphenol framework with restricted mobility [28, 40]. Together these perturbations establish Zn^2+^ as the central mediator converting a simple binary mixture into an ordered coordination network.

XPS analysis was performed to examine the elemental composition of ZnCA (Figure 2b). The survey spectrum showed signals corresponding to Zn, O, and C, confirming the incorporation of Zn into the nanoassembly. In the high‐resolution spectra, the C 1s signal was deconvoluted into components assigned to C–C/C═C (284.8 eV), C–O (286.6 eV), and C═O (287.8 eV), whereas the O 1s signal contained components associated with C═O (531.6 eV) and C–O/O–H species (533.2 eV). A weak Zn 2p signal was also detected; however, because of its limited signal‐to‐noise ratio, the Zn 2p binding energies, spin–orbit splitting, and zinc chemical state could not be determined reliably. Therefore, the Zn 2p spectrum is considered evidence of Zn incorporation rather than direct proof of Zn–O bond formation. Together with the changes observed in the complementary spectroscopic analyses, these results are consistent with interactions between Zn species and the oxygen‐containing groups of the organic ligands. Energy‐dispersive X‐ray spectroscopy and elemental mapping (Figure 2c, d) gave Zn 9.38, C 65.69, and O 24.66 at.%, consistent with a dense metal–phenolic network, with Zn, C, and O uniformly co‐localized across each sphere. This homogeneity argues against localized precipitation or surface adsorption, indicating that Zn^2+^ is incorporated as an integral structural node of ZnCA.

### 4.2 Enhanced Aqueous Dispersibility of ZnCA NPs

The preventive potential of polyphenols, particularly Cur, is fundamentally restricted by their extreme hydrophobicity and poor aqueous solubility [41]. Thus, we systematically evaluated the dispersibility of ZnCA in various physiologically and pharmaceutically relevant media (Figure 2e,f). In both acidic (pH 2.0) and neutral (pH 7.4) PBS, both the binary CA and coordinated ZnCA exhibited dramatically improved dispersibility compared to free Cur, which rapidly precipitated within 2 h. Interestingly, while no significant difference was observed between CA and ZnCA in the high‐ionic‐strength PBS environments, the superiority of the coordinated system became evident in deionized water (D‐water) and 1% DMSO. In these media, ZnCA demonstrated significantly higher colloidal stability and dispersibility retention than CA, which in turn outperformed free Cur. This enhanced performance in pure water and organic/aqueous mixtures suggests that the Zn^2+^ coordination‐driven assembly provides a more robust structural integrity and an optimized surface energy compared to the purely organic CA framework. The bridging effect of Zn^2+^ likely anchors the polyphenol ligands in a more stable, amorphous configuration that prevents self‐aggregation and facilitates more effective solvation [42, 43]. Collectively, these findings highlight that the coordination‐driven strategy not only achieves high metal loading but also yields a nanoplatform with superior dispersibility across diverse environments, a prerequisite for efficient gastrointestinal delivery and mucosal interaction in IBD therapy.

### 4.3 Thermal Stability, Environmental Resilience, and In Vitro Gastrointestinal Behavior of ZnCA NPs

**FIGURE 3:**
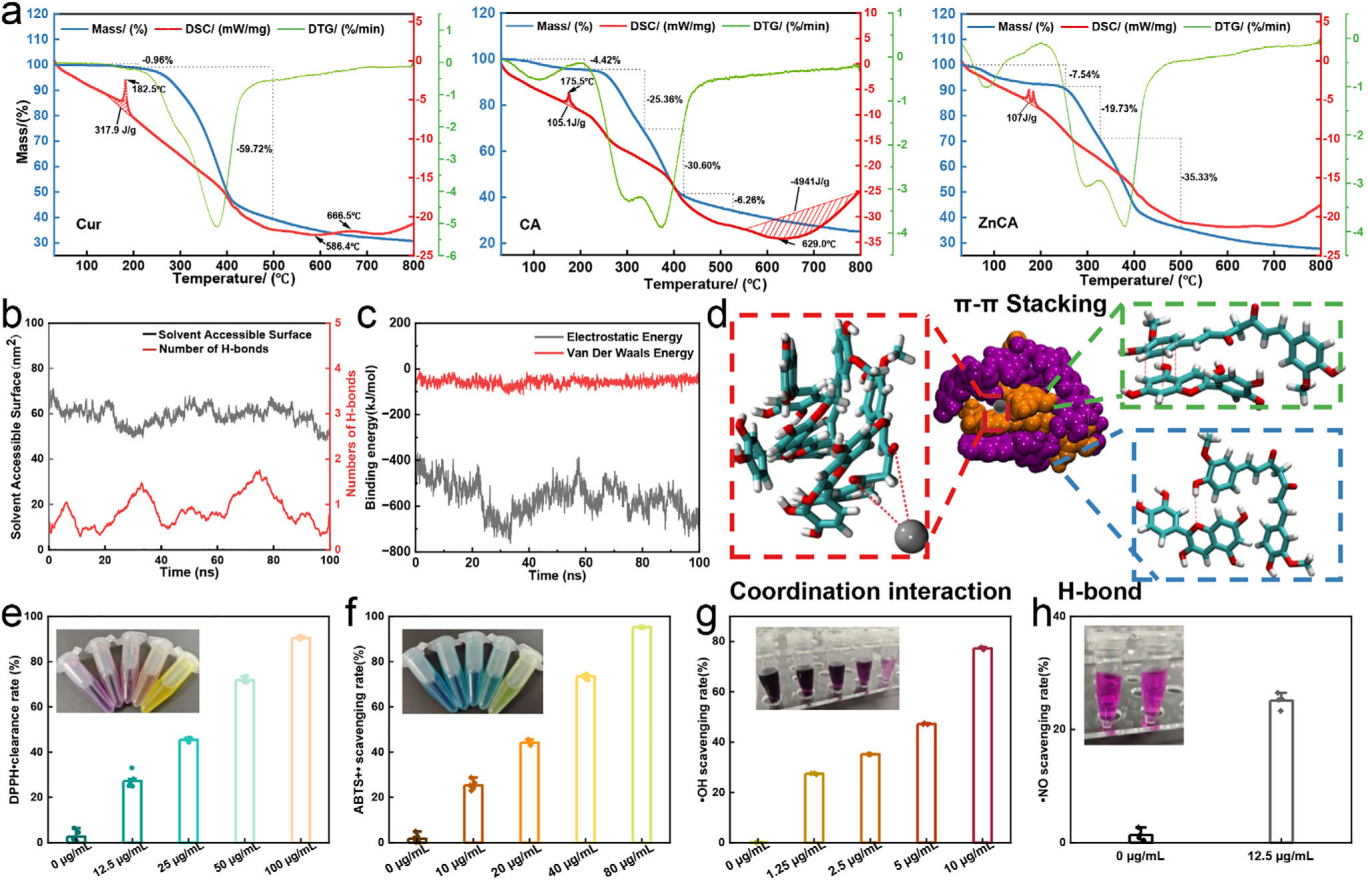
Thermal stability, molecular dynamics simulations, and broad‐spectrum RONS scavenging capacity of ZnCA nanoparticles. (a) Thermogravimetric analysis (TGA), derivative thermogravimetry (DTG), and differential scanning calorimetry (DSC) profiles of Cur, CA, and ZnCA. Molecular dynamics (MD) analysis of the ZnCA assembly over a 100 ns simulation: (b) solvent‐accessible surface area (SASA) and number of hydrogen bonds, and (c) van der Waals and electrostatic interaction energies. (d) Representative molecular configurations illustrating the coordination interactions, π–π stacking, and hydrogen bonding involved in ZnCA assembly. In vitro scavenging activities of ZnCA against different reactive oxygen and nitrogen species (RONS), including (e) DPPH•, (f) ABTS•+, (g) hydroxyl radicals (•OH), and (h) nitric oxide radicals (NO•). Insets show representative photographs of the corresponding colorimetric assays. Data in (e)–(h) are presented as mean ± SD (*n* ═ 6 for (e) and (f), *n* ═ 3 for (g), and *n* ═ 4 for (h)).

The structural robustness and phase transition behaviors of the as‐prepared nanoplatforms were evaluated by simultaneous TGA and DSC (Figure 3a). Free Cur exhibited a sharp and intense endothermic peak at 182.5 °C with a fusion enthalpy (Δ*H*_fusion_) of 317.9 J/g, characteristic of its highly ordered crystalline lattice. In contrast, the binary CA and coordinated ZnCA nanostructures displayed significantly suppressed and broadened endothermic signals at 175.5 and 174.6/183.5 °C, respectively. Notably, the Δ*H*_fusion_ of ZnCA (∼107 J/g) was markedly reduced compared to free Cur, suggesting that the coordination‐driven assembly with Zn^2+^ effectively disrupted the long‐range crystalline packing of the polyphenol ligands, facilitating a transition toward a more pharmacologically favorable amorphous or semi‐amorphous state.

The TGA and corresponding derivative thermogravimetry (DTG) curves revealed distinct pyrolytic patterns that differentiate the coordination complex from its precursors. At the initial heating stage (<120 °C), free Cur remained highly hydrophobic with a negligible weight loss of only 0.96%. Conversely, ZnCA exhibited a prominent initial weight loss of 7.54% (DTG peak at 82.1 °C), which was significantly higher than that of CA (4.42%). This increased mass loss is attributed to the release of surface‐adsorbed moisture and, more importantly, coordinated water molecules within the metal–phenolic network, consistent with the enhanced hydrophilicity and formation of the Zn^2+^‐mediated architecture. The subsequent thermal degradation of ZnCA followed a complex, multi‐stage kinetic process. While free Cur underwent a rapid, single‐step decomposition starting at ∼250 °C with a maximum degradation rate at 380 °C (DTG value of −5.09%/min), the ZnCA platform exhibited a more controlled mass loss profile. Specifically, ZnCA displayed sequential degradation steps: an initial weight loss of 19.73% at 296.1 °C, followed by a second stage at 379.1 °C, reaching a cumulative weight loss of 35.33% by 500 °C. The emergence of these distinct DTG sub‐peaks in ZnCA, as opposed to the singular peak of free Cur, reflects extensive structural integration and the formation of robust coordination bonds between Zn^2+^ and the Cur/Ant ligands. This coordination anchors the organic backbones, thereby modulating the thermal decomposition kinetics and conferring superior structural integrity under varying thermal environments. At the termination of the heating process (800 °C), the heat flow curves for both CA and ZnCA showed an upward trend, signifying the oxidative stabilization of the residual coordination framework. Collectively, these thermodynamic parameters confirm that the Zn^2+^‐mediated strategy transforms individual natural products into a stable, hydrated, and functionally integrated nanomedicine platform.

The environmental robustness of the ZnCA nanoplatform was systematically assessed against a range of external stressors, including thermal fluctuations, ultraviolet (UV) irradiation, and alkaline conditions (Figure S1a–c). While free Cur underwent rapid degradation across all tested parameters, ZnCA demonstrated markedly superior chemical stability and structural retention compared to both its free counterpart and the binary CA organic assembly. Notably, ZnCA preserved high structural integrity even after harsh 60‐min incubation at 90 °C or prolonged 24‐h UV exposure. This enhanced resilience is primarily attributed to the “cage effect” produced by the Zn^2+^‐mediated coordination network, which immobilizes the polyphenolic backbones and raises the energy barrier for thermal and oxidative decomposition [43]. Furthermore, the persistence of ZnCA in an alkaline microenvironment (pH 8.0) underscores the protective role of metal chelation in shielding the vulnerable β‐diketone and phenolate moieties from autoxidation and nucleophilic attack. Collectively, these findings demonstrate that the coordination‐driven strategy confers robust environmental resilience to natural bioactive compounds, preserving their structural integrity and biological function under physiological conditions.

To bridge the gap between environmental resilience and physiological applicability, the colloidal stability and in vitro release kinetics of the nanoplatform were sequentially evaluated under simulated gastrointestinal transit conditions (Figure S1d,e). Dynamic light scattering profiles demonstrated that ZnCA NPs possess robust structural stability against the contrasting pH environments of the gastrointestinal tract (Figure S1d). Following a 2‐h exposure to simulated gastric fluid (SGF, pH 1.2) and simulated intestinal fluid (SIF, pH 6.8), the nanoparticles sustained their colloidal stability, maintaining a relatively monodisperse, single‐peak distribution with well‐preserved Z‐average diameter and PDI values. This negligible structural perturbation or aggregation implies that the metal–phenolic crosslinking provides sufficient steric and electrostatic stabilization to withstand gastric acidity, preventing premature dissociation before reaching the targeted intestinal loci.

This protective effect was corroborated by the in vitro gastrointestinal release profiles (Figure S1e), evaluated via group‐specific normalization at 430 nm to eliminate co‐pigmentation interference. In acidic SGF, free Cur exhibited extensive release (∼55% at 72 h) due to its stability in acid and surfactant‐mediated solubilization. Conversely, both CA and ZnCA nano‐assemblies restricted leakage to ∼11%, demonstrating robust structural resilience. This minimal gastric release confirms that the hydrophobic domains and zinc‐coordination networks successfully prevent premature release in the stomach. Upon transitioning to SIF (pH 6.8), free Cur showed a deceptively low apparent release (∼13% at 72 h), a degradation artifact caused by polyphenol instability in neutral media. In contrast, ZnCA nanoparticles achieved a responsive, sustained release (∼37%), significantly outperforming both CA (∼28%) and free Cur. This enhanced dissolution confirms that the coordination‐induced quasi‐amorphous state lowers the crystalline lattice energy barrier, while the nano‐matrix shields the encapsulated cargo from rapid degradation [17, 33]. Collectively, these biomimetic evaluations demonstrate that the carrier‐free ZnCA nanoplatform couples transit stability with responsive target‐site dissolution, fulfilling a key prerequisite for oral nanomedicine delivery.

### 4.4 Molecular Dynamics Simulation of ZnCA Self‐assembly

To gain molecular‐level insights into the coordination‐driven self‐assembly of ZnCA, explicit‐solvent molecular dynamics (MD) simulations were performed over a 100 ns trajectory (Figure 3b–d). The ZnCA system, comprising Zn^2+^, Cur, and Ant, underwent an initial rapid association followed by dynamic structural rearrangement. As shown by the solvent‐accessible surface area (SASA) profile (Figure 3b), the SASA decreased sharply from approximately 70 to 50 nm^2^ during the first 35 ns, indicating rapid compaction of the initially dispersed components and the formation of a more condensed assembly. Analysis of the nonbonded interaction energies provided further insight into the forces contributing to this process (Figure 3c). The electrostatic interaction energy decreased from approximately −400 to −800 kJ/mol within the first 35 ns, suggesting that electrostatic attraction between Zn^2+^ and the oxygen‐containing groups of Cur and Ant played an important role in driving their initial association. By comparison, the van der Waals interaction energy fluctuated within a narrower range of approximately 0 to −100 kJ/mol, indicating a smaller but persistent contribution from short‐range intermolecular interactions [28]. After 35 ns, the electrostatic interaction energy partially recovered and subsequently fluctuated around −600 kJ/mol, while the SASA remained within approximately 50–60 nm^2^. These changes reflect the continued dynamic rearrangement of the assembled structure after its initial compaction [43, 44].

The number of intermolecular hydrogen bonds also varied throughout the simulation, with pronounced increases at approximately 35–40 and 70–80 ns (Figure 3b). These fluctuations indicate continuous reorientation of Cur and Ant molecules within the assembly, allowing the hydrogen‐bonding network to undergo dynamic reconstruction. Representative molecular configurations further revealed the coexistence of Zn^2+^–ligand coordination, π–π stacking, and hydrogen‐bonding interactions within the assembled system (Figure 3d). Collectively, the MD simulations suggest that ZnCA self‐assembly is governed by the cooperative contribution of electrostatic attraction, coordination interactions, π–π stacking, and hydrogen bonding [32, 43]. These results provide a molecular‐level explanation for the formation and dynamic stabilization of the multicomponent ZnCA architecture, consistent with its experimentally observed structural characteristics.

### 4.5 Broad‐Spectrum RONS‐Scavenging Capacity of ZnCA NPs

The fundamental mechanism of antioxidants involves the donation of hydrogen atoms or electrons to neutralize free radicals, thereby mitigating oxidative damage [45]. In this work, the broad‐spectrum RONS scavenging capacity of ZnCA NPs was assessed using diverse radical models (Figure 3e–h). As depicted in Figure 3e, the characteristic UV–vis absorbance peak of DPPH• at 517 nm was significantly attenuated upon the addition of ZnCA NPs, with the scavenging rate escalating from 27.21% at 12.5 µg/mL to 90.40% at 100 µg/mL. This spectral reduction was clearly manifested by a distinct visual transition from deep purple to yellow, indicating the progressive neutralization of the radicals. Similarly, ZnCA NPs exhibited a concentration‐dependent decolorization of ABTS•^+^ at 734 nm (Figure 3f), achieving a near‐complete clearance of 95.16% at a concentration of 80 µg/mL.

Given that hydroxyl radicals (•OH) are among the most reactive and damaging ROS in biological systems [46], the •OH‐scavenging activity of ZnCA NPs was further evaluated using the salicylic acid assay. ZnCA NPs markedly suppressed the formation of the colored products generated by the reaction between salicylic acid and •OH (Figure 3g), achieving a scavenging activity of 77.26% at 10 µg/mL. In addition to scavenging ROS, ZnCA NPs exhibited activity against the representative reactive nitrogen species nitric oxide (NO•), with a scavenging activity of 25.11% at 12.5 µg/mL (Figure 3h). Collectively, these results demonstrate that ZnCA NPs can scavenge multiple representative ROS and RNS in vitro, supporting their potential to alleviate oxidative and nitrosative stress in the inflamed colonic microenvironment.

### 4.6 In Vivo Biodistribution and Biosafety Evaluation

**FIGURE 4:**
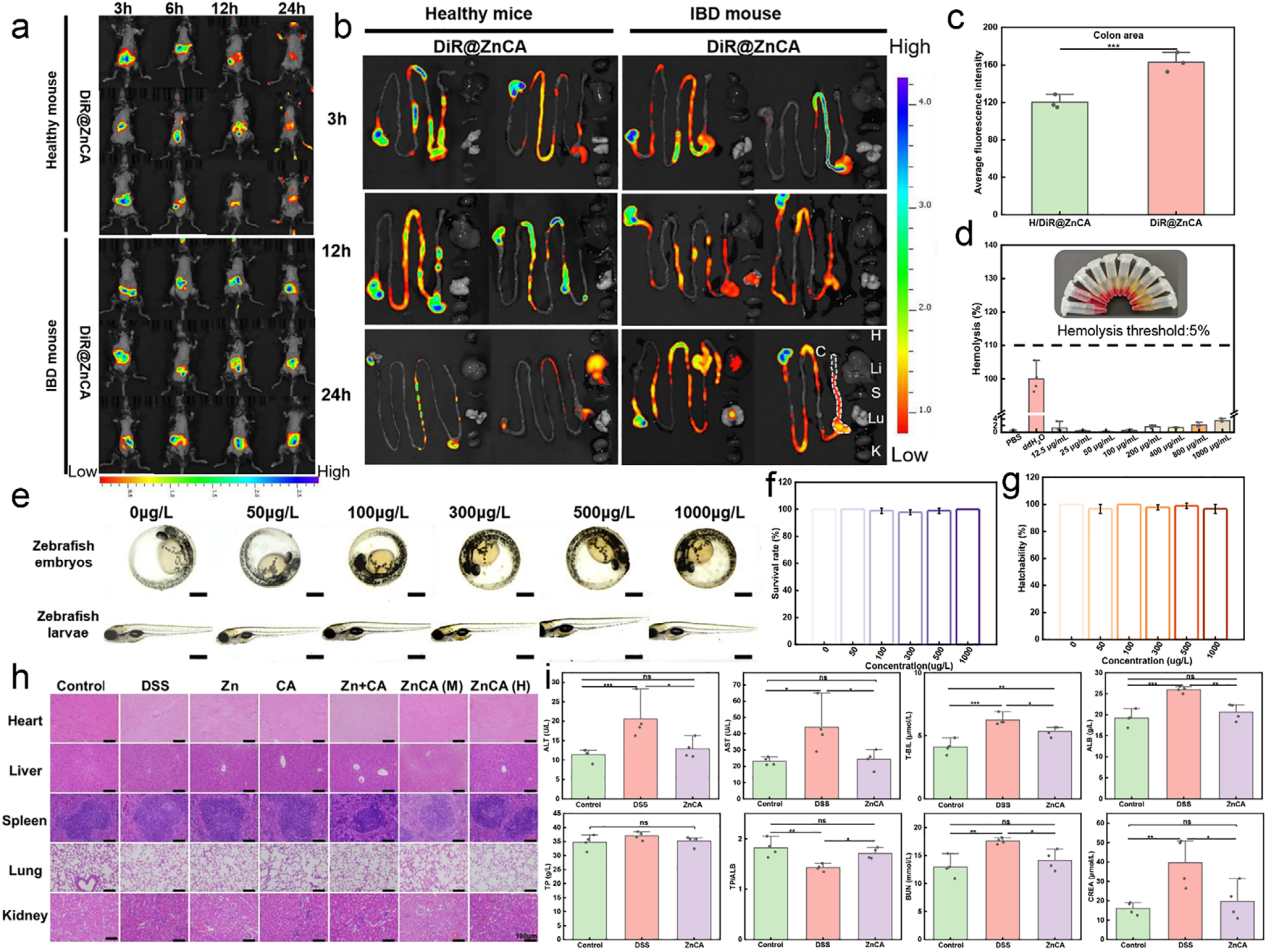
In vivo biodistribution and systemic biosafety evaluation of ZnCA. (a) Representative in vivo fluorescence images of healthy and IBD mice captured at scheduled intervals (3, 6, 12, and 24 h) post‐oral administration of DiR@ZnCA. (b) Ex vivo fluorescence imaging of major systemic organs (H: heart, Li: liver, S: spleen, Lu: lung, K: kidney) and the gastrointestinal tract (C: colon) captured at scheduled intervals (3, 12, and 24 h). (c) Quantitative fluorescence intensity of DiR signals in the colonic region of healthy and IBD mice (*n* ═ 3). (d) Hemolysis percentage of mouse red blood cells (mRBCs) incubated with ZnCA NPs (12.5–1000 µg/mL) (*n* ═ 4). The dashed line indicates the 5% hemolysis threshold. (e) Representative morphological snapshots of zebrafish embryos (36 hpf) and larvae (72 hpf) exposed to ZnCA NPs (0–1000 µg/L). Scale bar: 500 µm. (f) Survival rate and (g) hatching rate of zebrafish. For each concentration, 30 individuals were used per group, and the experiments were performed in triplicate (total *n* ═ 90 per group). (h) Histopathological H&E staining of the major organs harvested from mice in the Control, DSS, Zn, CA, Zn+CA, ZnCA(M), and ZnCA(H) groups (Scale bar: 100 µm). (i) Serum biochemical analysis of hepatic and renal function markers, including alanine aminotransferase (ALT), aspartate aminotransferase (AST), total bilirubin (T‐BIL), albumin (ALB), total protein (TP), the total protein‐to‐albumin ratio (TP/ALB), blood urea nitrogen (BUN), and creatinine (CREA) from mice in the Control, DSS, and ZnCA (H) groups (*n* ═ 4). Data are expressed as mean ± SD. *p < 0.05, **p < 0.01, ***p < 0.001. ns indicates no significant difference compared to the Control group.

An ideal nanoplatform for IBD should exhibit prolonged retention at the inflamed site to maximize efficacy while minimizing systemic off‐target effects. We utilized near‐infrared DiR‐labeled DiR@ZnCA nanoparticles to track their gastrointestinal transit and biodistribution in both healthy and IBD mice (Figure 4a). Following oral administration, DiR@ZnCA exhibited prolonged fluorescence retention in the abdominal region of both groups. The IBD mouse group demonstrated a persistent signal up to 24 h, suggesting an extended residence time of the formulation‐associated dye within the intestinal tract. This sustained and localized abdominal signal profile underscores the favorable transit kinetics and retention of the nanoparticle‐associated dye. Ex vivo imaging of the dissected organs further verified this preferential gastrointestinal localization (Figure 4b). The fluorescence signals were confined within the gastrointestinal tract, while major systemic organs (heart, liver, spleen, lung, and kidney) generally exhibited negligible background accumulation, with only minor fluorescence occasionally observed in individual liver or lung samples. Specifically, the colonic fluorescence intensity of the DiR@ZnCA group in IBD mice was significantly higher than that in the healthy counterparts (Figure 4c, *p* < 0.001). While macroscopic near‐infrared imaging primarily reflects the localization of the nanoparticle‐associated dye and cannot completely decouple the signals of fully intact nanostructures from locally partitioned dye, this distinct retention profile underscores a significant formulation‐mediated mucosal association. This enhanced signal retention is likely driven by the “EPR‐like” effect in the inflamed intestinal mucosa (ELVIS effect) and the optimized mucoadhesive properties of the zinc‐coordinated phenolic network, which together facilitate deep penetration and entrapment within the microenvironment of the inflamed colon [47].

For any orally administered nanomedicine, particularly in the context of IBD where the intestinal epithelial barrier is compromised, a comprehensive assessment of biosafety is essential. We first evaluated the hemocompatibility of ZnCA NPs using an in vitro hemolysis assay (Figure 4d). Across a wide concentration range (12.5–1000 µg/mL), ZnCA NPs induced negligible hemoglobin release. Even at the highest concentration tested (1000 µg/mL), the hemolysis rate remained below the 5% hemolysis threshold, indicating favorable hemocompatibility under the tested conditions. The environmental and developmental toxicity was subsequently evaluated using a zebrafish model (Figure 4e). Using 30 individuals per concentration in triplicate (total *n* ═ 90), we observed that ZnCA NPs (up to 1000 µg/L) caused no developmental abnormalities or mortality, with survival (Figure 4f) and hatching rates (Figure 4g) comparable to the control group. Furthermore, the systemic biocompatibility of ZnCA NPs was validated in vivo using a murine model. Histopathological analysis via H&E staining revealed that the structural architecture of major metabolic and filtration organs (heart, liver, spleen, lung, and kidney) in the ZnCA‐treated groups [both ZnCA(M) and ZnCA(H)] remained intact, showing no signs of inflammation, tissue lesions, or focal necrosis compared to the healthy control (Figure 4h). This was supported by quantitative comparisons of relative organ weights, which showed no significant differences (ns) across all experimental groups, ruling out gross material‐induced hypertrophy or atrophy (Figure S2a–d). To quantitatively evaluate systemic strain, a panel of serum biochemical markers was monitored (Figure 4i). In the DSS‐challenged group, serum levels of alanine aminotransferase (ALT), aspartate aminotransferase (AST), total bilirubin (T‐BIL), blood urea nitrogen (BUN), and creatinine (CREA) were significantly elevated, reflecting severe acute multiorgan stress. Meanwhile, while the TP level showed no significant change, ALB was notably elevated, leading to a significant decrease in the TP/ALB ratio compared to the healthy control group. Crucially, oral administration of ZnCA NPs effectively restored these biomarkers to levels statistically indistinguishable from the healthy control group. This comprehensive normalization of hepatic and renal functional parameters, coupled with the pristine histological profiles, demonstrates a favorable safety profile and confirms the low toxicological risk of the ZnCA nanoplatform under the evaluated experimental conditions. Nevertheless, certain limitations inherent to the current toxicological evaluation must be acknowledged. While these findings sufficiently validate the biosafety of ZnCA NPs within the evaluated dosing window of acute colitis, they do not fully extrapolate to long‐term chronic outcomes. Specifically, this study lacks quantitative, longitudinal tracking of elemental zinc levels in off‐target tissues over extended post‐treatment recovery periods, as well as precise mass‐balance quantification across excretion pathways. A comprehensive investigation into long‐term pharmacokinetics, definitive mass‐balance profiling, and chronic elemental accumulation represents a critical next step to fully delineate the safety margins of this self‐assembled platform during subsequent preclinical and industrial development phases.

### 4.7 ZnCA NPs Attenuate DSS‐Induced Colitis in Zebrafish Model

**FIGURE 5:**
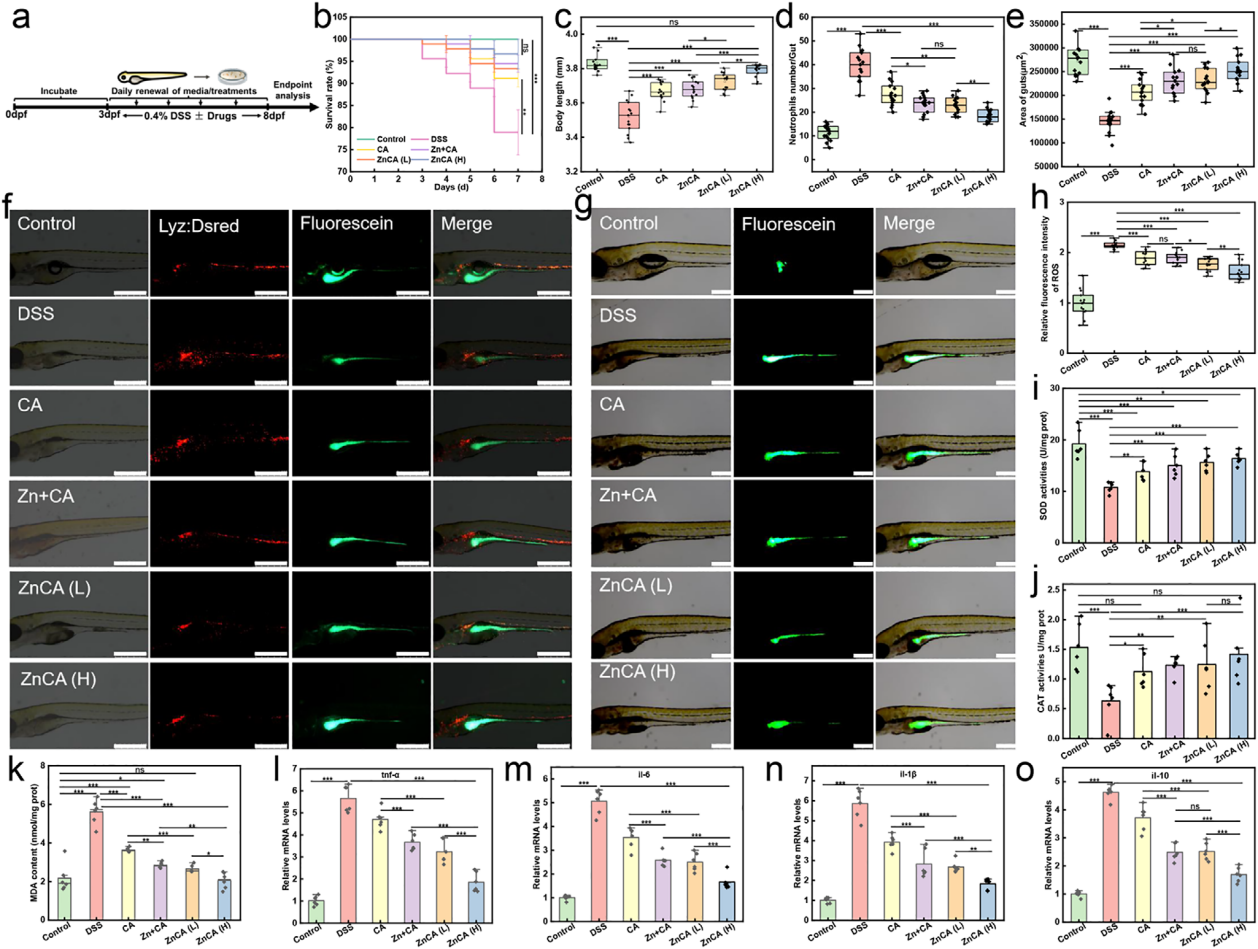
ZnCA coordination self‐assembled nanoparticles alleviate DSS‐induced colitis in zebrafish by suppressing neutrophil infiltration and oxidative stress. (a) Schematic illustration of the experimental protocol for DSS‐induced colitis modeling and drug treatments in zebrafish larvae. (b) Survival rates of zebrafish larvae in different treatment groups (Control, DSS, CA, Zn+CA, ZnCA (L), and ZnCA (H)) monitored from 3 to 8 dpf (*n* ═ 30 per group, three independent replicates). (c) Statistical analysis of the body length of zebrafish larvae at 8 dpf (*n* ═ 15). Quantitative analysis of (d) the number of infiltrated neutrophils in the intestine and (e) the intestinal area (µm^2^) calculated using Image‐Pro Plus 6.0 (*n* ═ 15). (f) Representative fluorescence microscopy images of *Tg*(*lyz*:*DsRed2*) transgenic zebrafish larvae at 8 dpf. Neutrophil migration is shown in red (Lyz:DsRed channel), and intestinal morphology is visualized by fluorescein staining (green channel). Scale bars, 500 µm. (g) Representative fluorescence images showing reactive oxygen species (ROS) levels in the intestine, detected by DCFH‐DA staining (green). Scale bars, 500 µm. (h) Quantification of the relative fluorescence intensity of ROS in the intestinal region analyzed using Image‐Pro Plus 6.0 (*n* ═ 15). Biochemical analysis of oxidative stress indicators including (i) SOD activity, (j) CAT activity, and (k) MDA content in zebrafish larvae (*n* ═ 50 per group, three independent replicates). Relative mRNA expression levels of inflammatory cytokines (l) tnf‐α, (m) *il‐6*, (n) *il‐1β*, and (o) *il‐10* determined by RT‐qPCR (*n* ═ 30 per group, three independent replicates). Data in (c), (d), (e), and (h) are presented as box plots. Data in (i)–(o) are presented as mean ± SD. Statistical significance was analyzed using one‐way ANOVA. *p < 0.05, **p < 0.01, ***p < 0.001 vs the DSS group. For all comparative treatments, the administered dosages of ZnCA (M), CA, and Zn+CA were normalized to ensure strict equivalence of the respective active ingredients (Cur, Ant, and Zn) across all groups.

To provide a preliminary in vivo validation of the formulation, the protective efficacy of ZnCA NPs was first evaluated using a DSS‐induced zebrafish model. This early‐stage screening platform captures fundamental phenotypic features of acute tissue injury, including reduced survival and growth impairment (Figure 5a). As illustrated in the Kaplan–Meier survival curves (Figure 5b), the DSS‐challenged group exhibited a precipitous decline in survival rate to approximately 83.3% by 8 dpf (*p* < 0.001). Notably, while the binary CA system and the physical mixture Zn+CA provided moderate protection, the ZnCA treatment groups—particularly the high‐dose group ZnCA (H)—exhibited a survival rate nearly indistinguishable from the Control group. This systemic recovery was further corroborated by the restoration of larval body length (Figure 5c). DSS exposure induced significant growth retardation (3.51 ± 0.09 mm) compared to the healthy control (3.83 ± 0.05 mm). Interestingly, although Zn+CA (3.69 ± 0.06 mm) showed a slight improvement over the binary CA (3.66 ± 0.06 mm), its efficacy was significantly lower than that of the coordination‐assembled ZnCA (L) (3.73 ± 0.05 mm) at the same zinc and polyphenol dosage. This disparity highlights that ZnCA's superior protection is not merely additive but stems from the coordination‐driven self‐assembly, which likely enhances the stability and localized accumulation of the polyphenols within the inflamed gut environment. Furthermore, ZnCA (H) exhibited a dose‐dependent restorative effect, almost completely reversing the growth deficit induced by DSS.

A hallmark of IBD is the compromise of the intestinal mucosal barrier and the subsequent influx of inflammatory cells [48, 49]. We utilized Tg(lyz:DsRed2) transgenic zebrafish to visualize neutrophil recruitment and fluorescein staining to assess intestinal morphology (Figure 5d–f). In the DSS‐challenged zebrafish, the intestinal architecture suffered catastrophic damage, characterized by a significant contraction of the gut area to 147.2 ± 23.3 × 10^3^ µm^2^, compared to 271.7 ± 28.1 × 10^3^ µm^2^ in the healthy control. This structural collapse was tightly coupled with a massive recruitment of neutrophils to the intestinal tract (39.7 ± 7.1 cells/gut), indicating a state of hyper‐inflammation and compromised barrier function (Figure 5d,e) [50, 51]. The administration of ZnCA coordination‐assembled nanoparticles effectively decoupled the cycle of mucosal damage and immune cell infiltration. Notably, while the binary CA system and the physical mixture Zn+CA provided partial restoration of the gut area (208.7 ± 21.9 × 10^3^ and 229.3 ± 26.5 × 10^3^ µm^2^, respectively), the coordination‐assembled ZnCA (L) exhibited a superior capacity for barrier preservation (235.4 ± 23.7 × 10^3^ µm^2^), effectively suppressing neutrophil transmigration to 22.9 ± 3.4 cells/gut. This functional superiority—particularly the difference between Zn+CA and ZnCA (L) at the same elemental dosage—suggests that nanostructural integrity is pivotal, as it likely enhances the localized retention and sustained release of polyphenols within the inflamed gut. The high‐dose group, ZnCA (H), achieved the most robust protective outcome, restoring the intestinal area to 251.3 ± 23.8 × 10^3^ µm^2^ and curbing neutrophil counts to 18.7 ± 2.5 cells/gut. These results demonstrate that ZnCA NPs act as a multi‐functional protective shield: they not only preserve the physical integrity of the mucosal barrier to prevent further DSS‐induced erosion but also modulate the underlying inflammatory signaling that drives pathological neutrophil recruitment.

Oxidative stress is a primary driver of mucosal damage in colitis. As shown by DCFH‐DA staining (Figure 5g,h), DSS induction triggered a massive surge in intestinal ROS levels. While all treatment groups showed some antioxidant capacity, the ZnCA nanoparticles demonstrated a superior ability to quench ROS. This is attributed to the enhanced efficacy resulting from the coordination between the polyphenol ligands (curcumin and anthocyanin) and the zinc ions, the latter of which serve as a critical cofactor for antioxidant enzymes. Biochemical analysis confirmed that the ZnCA nanoplatform effectively preserves intestinal redox equilibrium. As shown in Figure 5i,j, DSS treatment severely compromised the endogenous antioxidant defense, characterized by depleted SOD and CAT activities. However, ZnCA administration elicited a robust, dose‐dependent reactivation of these enzymes. Notably, the coordinated ZnCA (L) group exhibited superior efficacy in restoring enzyme activities compared to the physical mixture (Zn+CA) and free CA, underscoring the importance of the coordination assembly in enhancing the bioavailability and performance of the components. Most remarkably, high‐dose ZnCA treatment fully reinstated SOD and CAT activities to levels statistically indistinguishable from healthy controls (e.g., CAT recovery to 1.41 ± 0.49 U/mg prot vs Control 1.52 ± 0.35 U/mg prot). This enzymatic restoration translated directly into the suppression of oxidative damage. The content of MDA, a marker of lipid peroxidation, was sharply elevated in the DSS group but was effectively mitigated by ZnCA treatment (Figure 5k). Consistent with the enzyme profiles, the ZnCA (H) group normalized MDA levels (2.06 ± 0.40 nmol/mg prot) to baseline values (2.17 ± 0.72 nmol/mg prot), confirming that the nanoplatform not only scavenges ROS but fundamentally rehabilitates the oxidative microenvironment.

To further investigate the molecular mechanisms underlying the ameliorative efficacy of the nanomedicine, we evaluated the mRNA expression profiles of canonical inflammatory cytokines. Exposure to DSS elicited a robust systemic inflammatory response, evidenced by the significant transcriptional upregulation of pro‐inflammatory mediators, including *tnf‐α*, *il‐6*, and *il‐1β* (Figure 5l–n). Notably, at an equivalent polyphenol dosage, the ZnCA (L) group demonstrated superior suppressive effects on these transcripts compared to both the CA complex and the Zn+CA physical mixture. This confirms that the metal–polyphenolic coordination is pivotal for augmenting the stability and bioavailability of the phytochemical ligands, thereby optimizing their immunomodulatory potency. Furthermore, ZnCA intervention exhibited a clear dose‐dependent inhibitory profile, with the ZnCA (H) group achieving the most profound attenuation of inflammatory signaling, effectively returning the expression levels toward a basal physiological state. Interestingly, the expression of the anti‐inflammatory cytokine *il‐10* was also markedly elevated in the DSS‐challenged group (Figure 5o). Rather than indicating a protective benefit, this paradoxical rise typically reflects a compensatory counter‐regulatory mechanism in response to overwhelming acute inflammation. Subsequent administration of ZnCA, particularly at the higher dosage, resulted in a significant reduction of *il‐10* levels back to homeostatic baseline [50, 52]. This trend indicates that by effectively neutralizing the primary inflammatory stimuli and facilitating mucosal repair, ZnCA mitigates the pathological requirement for such compensatory anti‐inflammatory surges. Collectively, these molecular findings underscore the potential of ZnCA as a promising nano‐intervention for reestablishing immune homeostasis in the context of IBD.

### 4.8 In Vivo Preventive Efficacy Against DSS‐Induced Colitis

**FIGURE 6:**
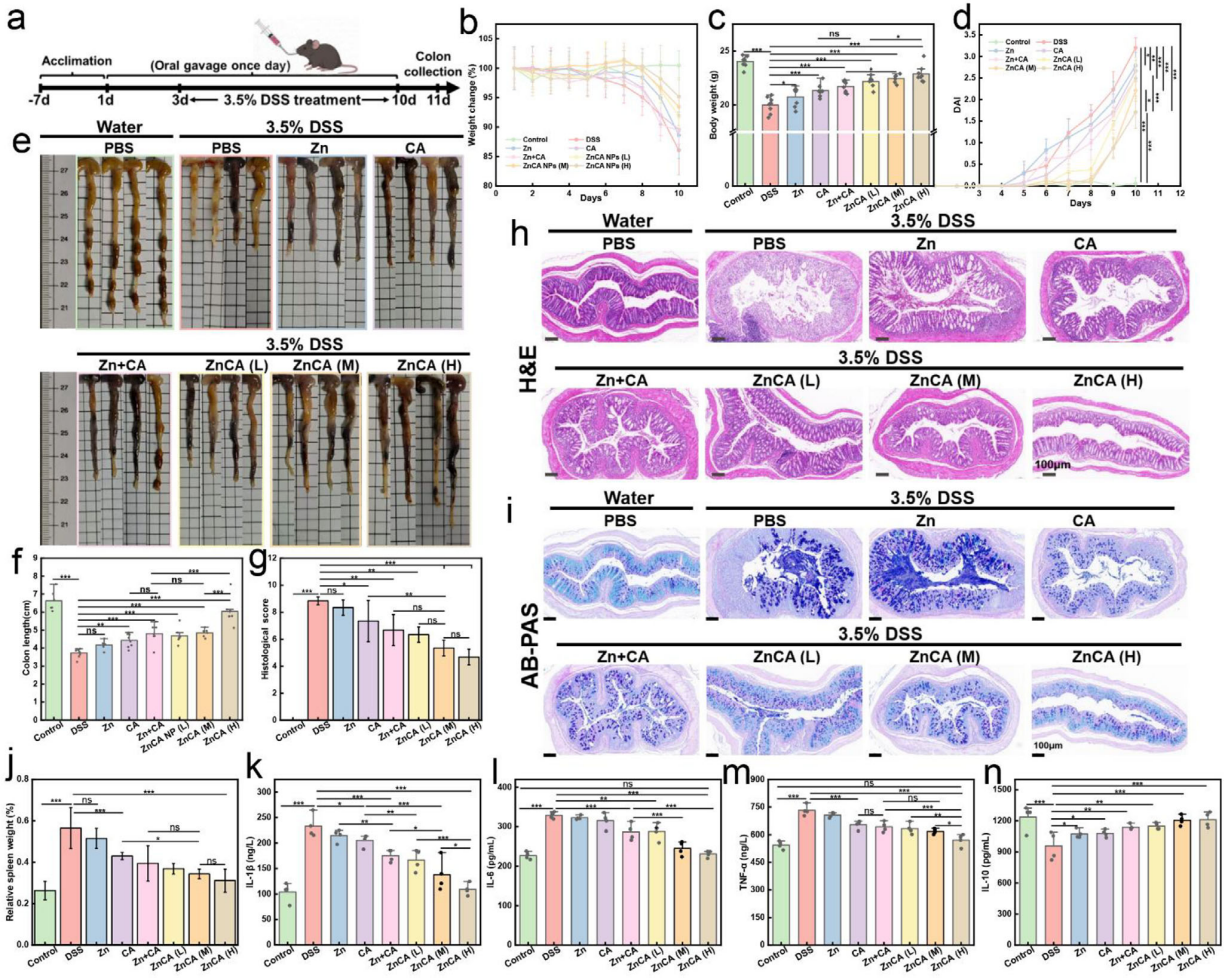
Protective efficacy of ZnCA nanoparticles against DSS‐induced colitis in mice. (a) Schematic illustration of the experimental design for the IBD model, showing the timeline for acclimation, prophylactic drug administration (3 days), and DSS exposure (7 days). (b) Daily body weight changes relative to day 0 (%) and (c) final body weights of mice at day 10 (*n* ═ 7–8). (d) Disease activity index (DAI) scores monitored throughout the experimental period (*n* ═ 7–8). (e) Representative macroscopic photographs of colons from different groups (*n* ═ 4) and (f) quantitative analysis of colon lengths (*n* ═ 6–8). (g) Histological scores based on H&E staining (*n* ═ 3) and (h) representative H&E‐stained microscopic images of colon sections (scale bar ═ 100 µm). (i) Representative AB‐PAS‐stained images showing mucus secretion and goblet cell integrity (scale bar ═ 100 µm). (j) Relative spleen weights (spleen‐to‐body weight ratio, %). Levels of inflammatory and anti‐inflammatory cytokines in colonic tissues determined by ELISA: (k) IL‐1β, (l) IL‐6, (m) TNF‐α, and (n) IL‐10 (*n* ═ 4). Data are presented as mean ± SD. Statistical significance is indicated as *p < 0.05, **p < 0.01, ***p < 0.001, and ns (not significant).

To rigorously evaluate the in vivo efficacy of the metal‐coordinated nanomedicines, we established a DSS‐induced colitis model in C57BL/6 mice (Figure 6a). Administration of 3.5% DSS induced a severe colitic phenotype, characterized by progressive weight loss and elevated DAI scores [53]. While monotherapies with carrier‐free CA nanocomplexes or free Zn^2+^ provided only moderate relief, ZnCA NPs demonstrated superior protective efficacy. Specifically, to elucidate the structural advantages of the coordination assembly, we compared the medium‐dose nanoparticles (ZnCA (M)) with a physical mixture (Zn+CA) containing equivalent amounts of Zn and CA.

As evidenced by the physiological monitoring (Figure 6b–d), treatment with ZnCA NPs effectively mitigated DSS‐induced deterioration. Crucially, at equivalent dosages, the ZnCA (M) group exhibited better maintenance of body weight and lower DAI scores than the Zn+CA physical mixture group. This suggests that the in situ coordination stabilizes the active components against gastrointestinal degradation more effectively than simple mixing does. Furthermore, the preventive effect of ZnCA NPs appeared strictly dose‐dependent; the low‐dose group (ZnCA (L)) provided partial protection, whereas the high‐dose group (ZnCA (H)) achieved maximal efficacy, restoring the final body weight and DAI to levels statistically comparable to the healthy Control group.

Macroscopic assessment of the colon and spleen provided compelling evidence of the superiority of the nanostructured formulation (Figure 6e,f,j). DSS treatment caused significant colon shortening due to severe inflammation. Treatment with ZnCA (M) resulted in significant preservation of colon length compared to the same dose of Zn+CA physical mixture, indicating that the coordinated nanoparticles facilitated better accumulation and retention of the bioactive agents in the inflamed tissue. Regarding systemic inflammation, DSS treatment induced marked splenomegaly (Figure 6j) [54, 55]. While free Zn^2+^ failed to alleviate this symptom (not significant vs DSS), all polyphenol‐containing groups (CA, Zn+CA, and ZnCA NPs) significantly inhibited splenomegaly (*p* < 0.01 or *p* < 0.001). Most notably, a direct comparison revealed that the ZnCA (M) group exerted a significantly stronger inhibitory effect on spleen enlargement than the carrier‐free CA group (*p* < 0.05). This result underscores that the coordination of CA with zinc ions not only serves a structural role but actively potentiates the systemic anti‐inflammatory efficacy beyond that of the carrier‐free formulation.

Histological examination using H&E and AB‐PAS staining confirmed the mucosal protective mechanism (Figure 6g–i). The DSS and Zn+CA groups displayed evident epithelial erosion and crypt distortion. In contrast, the ZnCA (M) group exhibited preserved crypt architecture and reduced inflammatory infiltration, and was superior to the physical mixture. The ZnCA (H) group showed the most intact mucosal structure, with histological scores significantly lower than those of the ZnCA (M) group. Moreover, AB‐PAS staining highlighted that administration of ZnCA NPs, particularly at medium and high doses, effectively preserved goblet cells and mucin secretion, which are critical for intestinal barrier integrity. At the molecular level, the regulation of inflammatory cytokines underscored the enhanced efficacy of the metal–phenolic coordination (Figure 6k–n). The DSS challenge triggered a “cytokine storm” with elevated IL‐1β, IL‐6, and TNF‐α. Comparative analysis revealed that ZnCA (M) exerted a stronger inhibitory effect on these pro‐inflammatory mediators than the equivalent Zn+CA mixture, likely due to enhanced cellular uptake facilitated by the nanoparticle size and surface properties. Notably, the ZnCA (H) group demonstrated the most profound anti‐inflammatory activity and significantly upregulated the anti‐inflammatory cytokine IL‐10. This active upregulation of IL‐10 in the murine colitis model, when contrasted with the post‐treatment reduction observed in zebrafish larvae, highlights the distinct spatiotemporal immune response strategies operating across different phylogenetic models. While the acute chemical injury in zebrafish triggers an immediate, systemic compensatory loop–manifesting as a transient emergency spike in *il‐10* [50, 52] that recedes once ZnCA NPs resolve upstream inflammatory triggers–the mammalian model represents a more protracted pathology characterized by localized immune exhaustion and severe suppression of endogenous IL‐10 production [56, 57]. Consequently, the preventive efficacy of ZnCA NPs in mice is distinguished by an active restoration of the colonic immune microenvironment, thereby enabling the upregulation of IL‐10 to drive multifaceted mucosal healing. Collectively, these results demonstrate that the protective efficacy of ZnCA NPs arises not only from the bioactive components themselves but specifically from the coordination‐driven nanostructure, which enhances bioavailability and efficacy in a dose‐dependent manner [58].

### 4.9 ZnCA NPs Restore Intestinal Barrier Integrity and Ameliorate the Inflammatory Microenvironment

**FIGURE 7:**
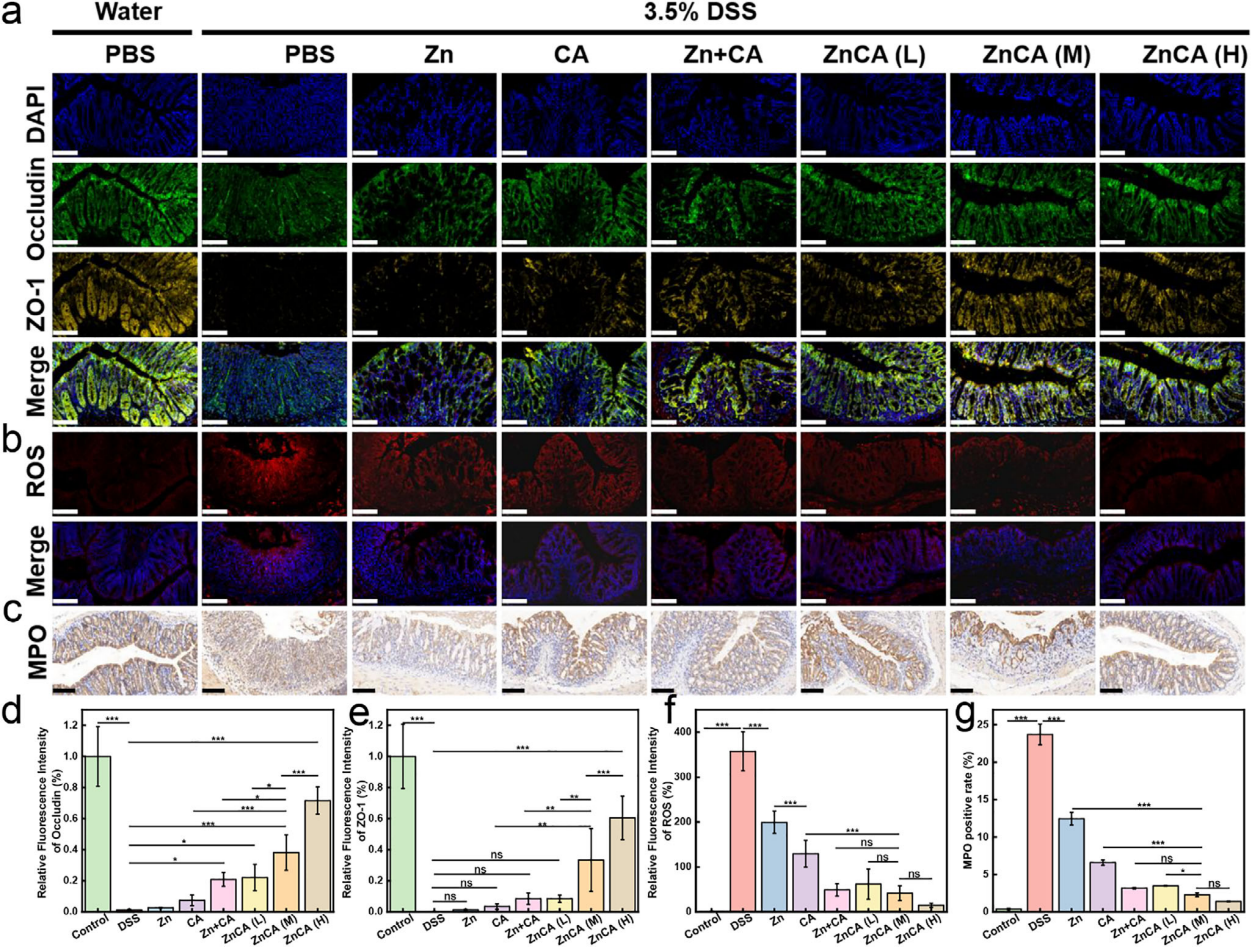
Effects of ZnCA NPs on intestinal barrier integrity, oxidative stress, and neutrophilic inflammation in a mouse model of DSS‐induced colitis. (a) Representative immunofluorescence images of tight junction proteins Occludin (green) and ZO‐1 (yellow) in colon tissues from different treatment groups. Nuclei were counterstained with DAPI (blue). (b) Representative immunofluorescence staining of ROS levels (red) in colon tissues. Nuclei were counterstained with DAPI (blue) in both (a) and (b). (c) Representative immunohistochemical staining images of MPO in colonic sections. Scale bars: 100 µm. Corresponding quantitative analysis performed using Image‐Pro Plus 6.0 (Media Cybernetics, Rockville, MD, USA): (d) relative fluorescence intensity of Occludin; (e) relative fluorescence intensity of ZO‐1; (f) relative fluorescence intensity of ROS; and (g) MPO‐positive rate. Data are presented as mean ± SD (*n* ═ 3). Statistical significance is indicated by *p < 0.05, **p < 0.01, ***p < 0.001 and ns (nonsignificant).

The disruption of the intestinal epithelial barrier, characterized by the loss of TJ proteins, is a critical hallmark of UC pathogenesis that facilitates bacterial translocation and aggravates inflammation [59, 60]. To evaluate the mucosal healing capacity of our engineered nanotherapeutics, we performed immunofluorescence analysis of two pivotal TJ proteins, Occludin and ZO‐1 (Figure 7a). In the DSS‐induced colitis group, the colonic tissues exhibited a severe collapse of the epithelial barrier, evidenced by a discontinuous staining pattern and markedly reduced fluorescence intensity of Occludin and ZO‐1 (Figure 7a).

Although CA and the Zn+CA physical mixture partially restored tight‐junction protein expression, free Zn ions produced only a limited improvement. Importantly, at equivalent component doses, ZnCA (M) achieved more pronounced restoration of tight‐junction integrity than CA and Zn+CA (Figure 7a,d,e). Quantitative analysis showed that the relative fluorescence intensity of Occludin was significantly higher in the ZnCA (M) group than in the Zn+CA group (*p* < 0.05, Figure 7d). ZO‐1 expression was also significantly increased in the ZnCA (M) group compared with the Zn+CA group (*p* < 0.01, Figure 7e). This suggests that the coordination‐driven assembly of ZnCA nanoparticles enhances the stability and retention of the active ingredients within the inflamed mucosa, thereby maximizing their formulation‐enabled biological effects [61, 62]. Furthermore, ZnCA treatment showed an overall dose‐dependent trend in restoring intestinal barrier integrity. Among the tested doses, ZnCA (H) produced the greatest recovery of intestinal barrier integrity. In the ZnCA (H) group, ZO‐1 exhibited a more continuous epithelial distribution resembling that observed in the healthy Control group (Figure 7a). Quantitative ZO‐1 fluorescence reached approximately 60% of the Control level and was significantly higher than that in the DSS group (*p* < 0.001, Figure 7e). These findings indicate that ZnCA nanoparticles effectively promote the reconstruction of the intestinal epithelial barrier, thereby potentially limiting the progression of barrier dysfunction‐associated inflammation [12].

Excessive accumulation of ROS in the inflamed colon acts as a primary driver of tissue damage and proinflammatory signaling pathways [58]. We therefore assessed the ROS‐scavenging capability of ZnCA in vivo (Figure 7b). The DSS group displayed intense red fluorescence, indicating severe oxidative stress. Consistent with the barrier restoration results, the ZnCA nanoparticles exhibited excellent antioxidative performance. Although the ROS signal tended to be lower in the ZnCA (M) group than in the Zn+CA group, the difference was not statistically significant (*p* > 0.05, Figure 7f). This observation is consistent with the ability of the coordination‐assembled formulation to retain the antioxidant activity of its constituent polyphenols, although the contribution of enhanced gastrointestinal stability requires further investigation. Among the dosage gradients, ZnCA (H) demonstrated superior antioxidative capacity, suppressing ROS levels to a near‐normal range (*p* < 0.001 vs DSS, Figure 7f). These findings demonstrate the potent in vivo antioxidant capacity of ZnCA nanoparticles.

To further investigate the anti‐inflammatory mechanism, we examined the expression of MPO, a marker of neutrophil infiltration (Figure 7c). High MPO levels in the DSS group confirmed severe neutrophilic inflammation. Treatment with ZnCA nanoparticles significantly reduced MPO‐positive cells in a dose‐dependent manner (Figure 7g). Although ZnCA (M) showed a lower MPO‐positive rate than the Zn+CA group, the difference was not statistically significant. ZnCA (H) produced the lowest MPO‐positive rate among the treatment groups, indicating effective attenuation of colonic neutrophilic inflammation [63, 64, 65]. Taken together, ZnCA NPs alleviated colonic oxidative stress and neutrophilic inflammation while promoting the restoration of the damaged intestinal mucosal barrier, as evidenced by reduced ROS accumulation and MPO‐positive staining and the recovery of Occludin and ZO‐1 expression and distribution. At equivalent component doses, ZnCA (M) exhibited more pronounced tight‐junction restoration than the Zn+CA physical mixture, highlighting the contribution of coordination‐driven nanoassembly to its intestinal barrier‐protective effects.

### 4.10 Transcriptomic Reprogramming and Suppression of Inflammatory Signaling by ZnCA

**FIGURE 8:**
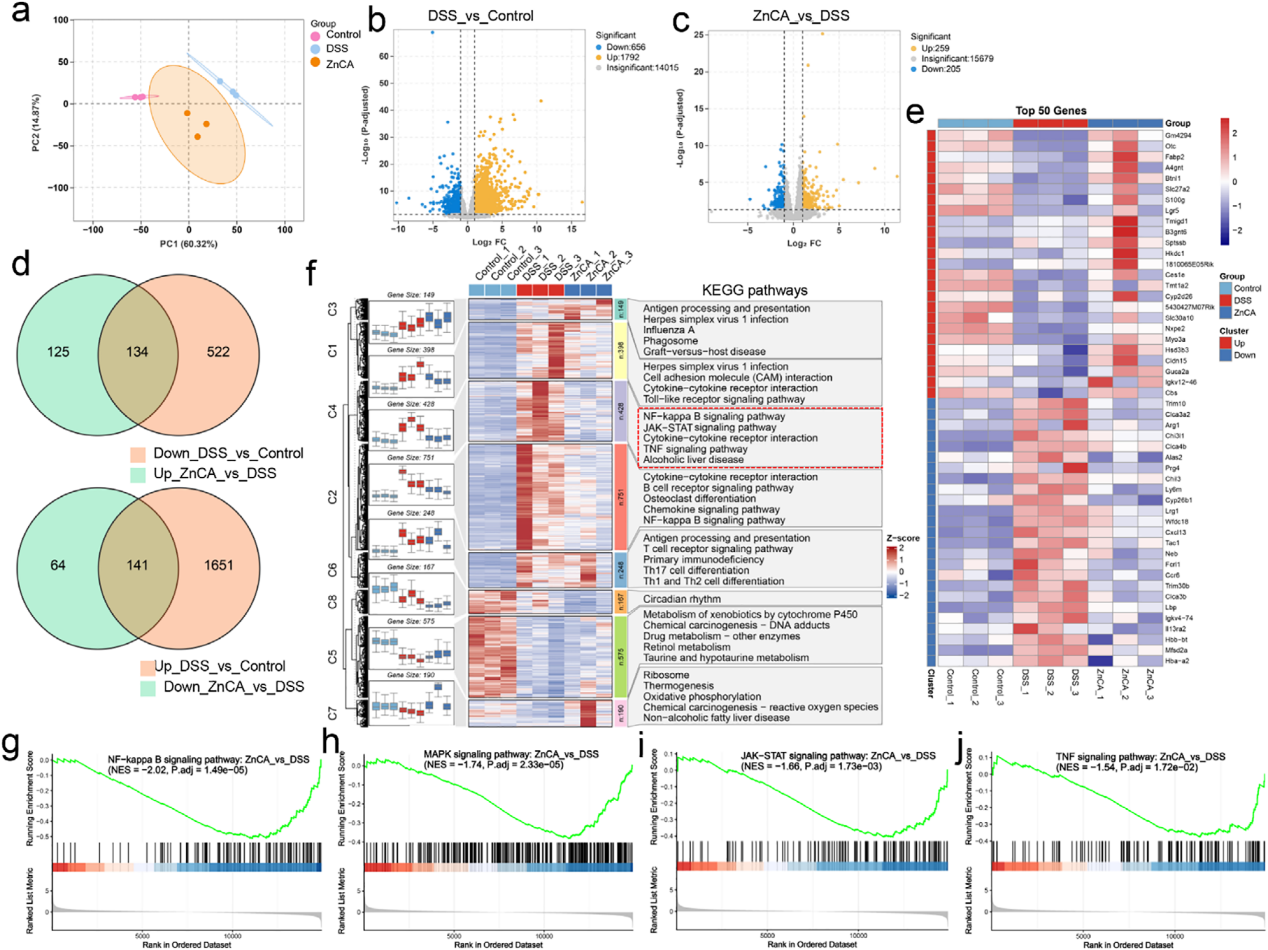
Transcriptomic profiling reveals the protective mechanism of ZnCA nanoparticles in reprogramming the inflammatory microenvironment. (a) Principal component analysis (PCA) plot displaying the spatial distribution and segregation of transcriptomic profiles from the Control, DSS, and ZnCA‐treated groups (*n* ═ 3 biologically independent samples). Volcano plots visualizing differentially expressed genes (DEGs) in the (b) DSS vs Control and (c) ZnCA vs DSS comparisons. Blue and orange dots represent significantly downregulated and upregulated genes, respectively (threshold: |Log_2_ fold‐change| ≥ 1, *P*_adj_ < 0.05). (d) Venn diagrams illustrating the specific intersection of DEGs. The overlap regions represent genes that were significantly dysregulated by DSS induction but effectively reversed by ZnCA treatment. (e) Hierarchical clustering heatmap of the top 50 DEGs between the ZnCA and DSS groups, demonstrating the distinct “reversal” of gene expression patterns by ZnCA treatment. (f) Integrated heatmap and KEGG pathway enrichment analysis classifying DEGs into functional clusters (C1–C8), highlighting the modulation of pathways related to immune regulation and inflammation. Gene Set Enrichment Analysis (GSEA) plots verifying the significant downregulation of key inflammatory signaling pathways in the ZnCA group compared to the DSS group: (g) NF‐κB, (h) MAPK, (i) JAK‐STAT, and (j) TNF signaling pathways. The normalized enrichment score (NES) and adjusted *P*‐value (*P*_adj_) are indicated in each panel.

To delineate the molecular landscape underlying the protective efficacy of ZnCA, we performed high‐throughput RNA sequencing to capture the gene expression changes in the gut tissue. Principal component analysis (PCA) unveiled a drastic transcriptomic shift, in which the DSS group segregated sharply from the Control group, indicative of a severe inflammatory perturbation. Notably, ZnCA treatment effectively reversed this pathological deviation, repositioning the global transcriptomic profile toward the Control group (Figure 8a). This transcriptomic reversion was further corroborated at the individual‐gene level by differential expression analysis. Whereas DSS induction caused widespread gene dysregulation (Figure 8b), ZnCA treatment markedly attenuated this response, yielding a more restrained set of DEGs (Figure 8c).

To define the specificity of this transcriptomic rescue, we examined the overlap of dysregulated genes using Venn diagrams (Figure 8d), which revealed clear bidirectional regulation. Specifically, 141 genes upregulated by DSS were significantly downregulated by ZnCA treatment, reflecting suppression of pathogenic factors; reciprocally, 134 genes repressed during colitis were restored toward physiological levels by ZnCA treatment. Hierarchical clustering of the top 50 DEGs (Figure 8e) further illustrated this protective pattern; pro‐inflammatory mediators (e.g., Trim10, Cxcl13, Wfdc18, Lbp) induced by DSS returned to near‐baseline levels after ZnCA administration, whereas genes critical for mucosal integrity and stem cell renewal (e.g., Lgr5, Otc) were successfully restored. We further mapped these ZnCA‐modulated genes to Kyoto Encyclopedia of Genes and Genomes (KEGG) pathways (Figure 8f), which classified the DEGs into distinct functional clusters (C1–C8). The analysis demonstrated that inflammatory clusters (e.g., Clusters C2 and C4), which are enriched in “NF‐kappa B signaling,” “TNF signaling,” and “JAK‐STAT signaling,” were strongly activated in the DSS group but markedly suppressed by ZnCA treatment. In parallel, clusters associated with physiological homeostasis (e.g., Cluster C5), such as “Metabolism of xenobiotics” and “Retinol metabolism,” were reactivated from a suppressed state, indicating that ZnCA reprograms the colonic transcriptome from a pro‐inflammatory toward a metabolically homeostatic state [5].

We interrogated the mechanistic alterations via Gene Set Enrichment Analysis (GSEA). The analysis revealed that ZnCA treatment orchestrated a synchronized blockade of the principal inflammatory signaling axes. Specifically, while DSS induction precipitated a catastrophic activation of the NF‐κB pathway (Figure S3a), ZnCA treatment functioned as an effective molecular brake, drastically suppressing this master regulator of inflammation (NES ═ −2.02, *P*_adj_ ═ 1.49 × 10^−5^; Figure 8g). This transcriptional silencing extended coordinately to the MAPK signaling cascade, which underwent a complete reversal following nanoparticle administration (NES ═ −1.74, *P*_adj_ ═ 2.33 × 10^−5^; Figures 8h and S3b). The protective impact was further substantiated by the broad‐spectrum inhibition of the JAK‐STAT (NES ═ −1.66; Figures 8i and S3c) and TNF signaling pathways (NES ═ −1.54; Figures 8j and S3d). Collectively, these data demonstrate that ZnCA nanoparticles do not merely affect isolated targets but modulate the core inflammatory signaling pathways established by DSS, thereby driving the pathological transcriptome back toward a homeostatic state [14].

### 4.11 Remodeling of the Gut Microbiota Composition by ZnCA Nanoparticles

**FIGURE 9:**
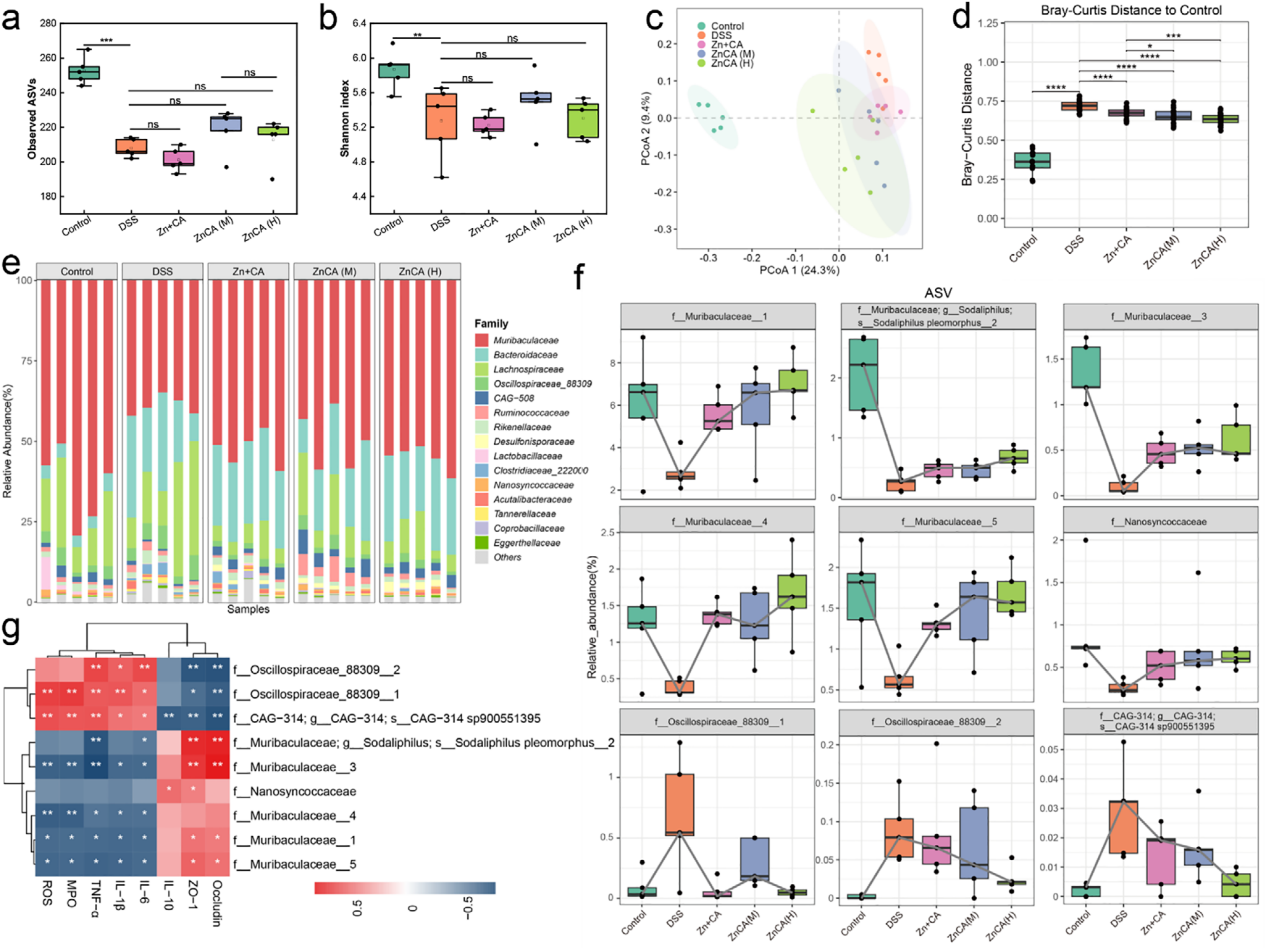
ZnCA nanoparticles remodel the gut microbiota landscape to attenuate DSS‐induced colitis in mice. Gut microbiota diversity analysis across different treatment groups: alpha diversity indices representing the richness and diversity, including (a) observed ASVs and (b) the Shannon index; beta diversity profiling illustrating the structural segregation of microbial communities, including (c) principal coordinates analysis (PCoA) plot based on Bray–Curtis distance and (d) boxplot of Bray–Curtis distance to the Control group. (e) Stacked bar chart displaying the taxonomic composition and relative abundance of dominant bacteria at the family level. (f) Relative abundances of representative differentially abundant bacterial ASVs modulated by ZnCA treatment, including ASVs assigned to Muribaculaceae and Oscillospiraceae__88309. (g) Spearman's correlation heatmap showing the associations between key microbial taxa and inflammatory/biochemical markers (ROS, MPO, TNF‐α, IL‐1β, IL‐6, IL‐10, ZO‐1, and Occludin). The color scale represents the Spearman correlation coefficient, where red indicates a positive correlation and blue indicates a negative correlation. Data are presented as mean ± SD (*n* ═ 5 biologically independent samples per group). Statistical significance was determined by one‐way ANOVA followed by Tukey's post hoc test. Group comparisons are indicated by brackets: *P < 0.05, **P < 0.01, ***P < 0.001, ****P < 0.0001, and ns (non‐significant).

To unravel the ecological mechanisms accompanying the protective efficacy of ZnCA, we characterized the gut microbiome landscape via 16S rRNA gene amplicon sequencing. DSS caused severe dysbiosis, marked by a distinct contraction of microbial diversity (Figure 9a,b) [62, 66]. Following intervention, although the overall microbial richness did not differ significantly from that in the DSS group, a clear dose‐dependent trend toward recovery was observed in the ZnCA (M) and ZnCA (H) groups, particularly in the number of observed ASVs. PCoA based on Bray–Curtis distance (Figure 9c) revealed that the microbial communities of ZnCA‐treated mice formed distinct clusters that shifted away from the DSS group and positioned closer to the Control, which was further quantified by the Bray–Curtis distance to the Control group (Figure 9d), showing ZnCA‐treated groups exhibited significantly smaller Bray–Curtis distances to the Control group than the DSS group did (*p* < 0.0001).

Taxonomic profiling at the family level (Figure 9e) further dissected the specific bacterial alterations driving this structural shift. The ameliorative impact of ZnCA was characterized by the preservation of beneficial commensals. Specifically, we observed a significant enrichment of multiple ASVs assigned to the family Muribaculaceae, a dominant carbohydrate‐degrading and SCFA‐producing family in the murine gut [67]. Several Muribaculaceae lineages, which were markedly depleted by DSS, exhibited a significant, dose‑dependent increase following treatment with ZnCA nanoparticles. Similarly, the abundance of f__Nanosyncoccaceae, which was suppressed under DSS‐induced colitis, also showed a clear restoration following ZnCA administration (Figure 9f). Conversely, ZnCA treatment effectively suppressed the expansion of potential pathobionts that bloomed during colitis. Specifically, members of the f_*_*Oscillospiraceae__88309 and f__CAG‐314; g__CAG‐314; s__CAG‐314 sp900551395 (Figure 9f) exhibited an aberrant expansion in the DSS group. Although Oscillospiraceae is frequently regarded as a health‐associated family, recent studies have revealed a significant increase in its abundance among patients with ulcerative colitis. This enrichment is closely linked to nicotinamide metabolic dysfunction and delayed intestinal transit, suggesting that Oscillospiraceae may exacerbate intestinal dysfunction under specific pathological conditions [68, 69]. ZnCA administration significantly inhibited this overgrowth, with ZnCA(H) achieving the most pronounced inhibitory effect, returning its abundance to levels comparable to those in the Control group.

The functional significance of these shifts was further validated by Spearman's correlation heatmap (Figure 9g). The Muribaculaceae ASVs and f__Nanosyncoccaceae preserved by ZnCA treatment were negatively correlated with pro‐inflammatory markers (ROS, TNF‐α, IL‐1β, IL‐6, and MPO) and positively correlated with barrier proteins (ZO‐1 and Occludin) as well as the anti‐inflammatory cytokine IL‐10, while the DSS‐enriched f__Oscillospiraceae_88309 and f__CAG‐314; g__CAG‐314; s__CAG‐314 sp900551395 showed strong positive correlations with oxidative stress and inflammatory cytokines. Collectively, these results demonstrate that ZnCA nanoparticles maintain intestinal integrity and modulate the microbiota balance.

### 4.12 Verification of the Microbiota‐Mediated Protection

**FIGURE 10:**
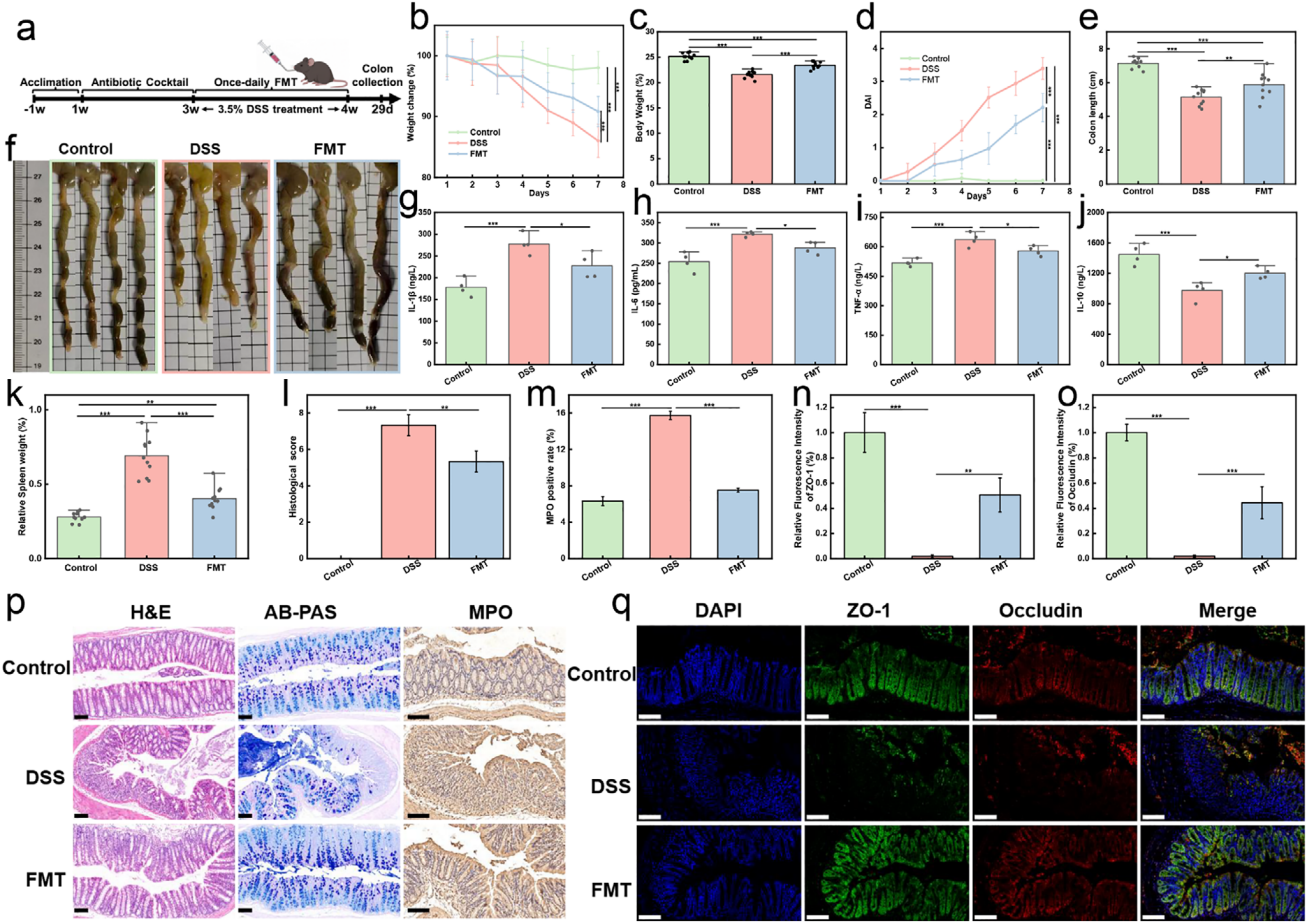
Fecal microbiota transplantation (FMT) transfers the protective benefits of ZnCA‐regulated microbiota to DSS‐induced colitis mice. (a) Schematic illustration of the experimental design and timeline for the FMT procedure. (b) Body weight changes and (d) disease activity index (DAI) scores of mice monitored throughout the modeling and treatment period (*n* ═ 10–11 per group). (c) Final body weight of mice at the time of sacrifice (*n* ═ 10–11). Assessment of colonic shortening: (e) quantitative analysis of colon length (*n* ═ 8) and (f) representative macroscopic images of colons. ELISA analysis of inflammatory cytokine levels in colonic tissues: (g) IL‐1β, (h) IL‐6, (i) TNF‐α, and (j) IL‐10 (*n* ═ 4). (k) Relative spleen weight (spleen index) quantification (*n* ═ 10–11). Quantitative analysis of histological damage and mucosal barrier integrity: (l) histological scores based on H&E staining (*n* ═ 3), (m) positivity rate of MPO (*n* ═ 3), and relative fluorescence intensity of (n) ZO‐1 and (o) Occludin (*n* ═ 3). (p) Representative microscopic images of colon sections stained with H&E, AB‐PAS, and immunohistochemical staining for MPO (scale bars ═ 100 µm). (q) Representative immunofluorescence staining images of tight junction proteins ZO‐1 (green) and Occludin (red) in colon tissues, with nuclei counterstained with DAPI (blue) (scale bars ═ 100 µm). Data are presented as mean ± SD. Statistical significance was determined by one‐way ANOVA followed by Tukey's post hoc test: *P < 0.05, **P < 0.01, and ***P < 0.001.

To determine whether the gut microbiota remodeled by ZnCA contributed to its protective effects, we performed a fecal microbiota transplantation (FMT) experiment. Fecal microbiota were collected from ZnCA‐treated donor mice after a three‐day washout period to minimize the potential carryover of residual nanoparticles and were subsequently transplanted into DSS‐treated recipient mice (Figure 10a). Recipients of the ZnCA‐modulated microbiota showed a clear improvement in colitis‐related symptoms, partially reproducing the protective effects observed following direct ZnCA treatment. Compared with the DSS group, mice receiving FMT experienced less body weight loss and maintained higher body weights throughout the experimental period (Figure 10b,c). Microbiota transfer also significantly reduced DAI scores (Figure 10d) and alleviated DSS‐induced colonic shortening (Figure 10e,f). These findings indicate that the microbiota remodeled by ZnCA can transfer part of its protective effect to recipient mice and therefore contributes to the alleviation of DSS‐induced colitis.

Beyond these phenotypic improvements, the transplanted microbiota was accompanied by a favorable modulation of the colonic immune microenvironment and systemic inflammatory status. At the systemic level, DSS‐induced splenomegaly was mitigated in the FMT recipients, as reflected by the normalized spleen size and spleen index (Figure 10k), indicating a reduction in systemic inflammation. Locally within the colonic tissue, the immunomodulatory effects associated with FMT were evaluated by measuring colonic cytokine levels via ELISA. The intervention was linked to a suppressed hypersecretion of key pro‐inflammatory cytokines, including IL‐1β, IL‐6, and TNF‐α (Figure 10g‐i), accompanied by upregulation of the anti‐inflammatory cytokine IL‐10 (Figure 10j). This shift suggests that the gut microbiota modulated by ZnCA may help reshape the intestinal milieu from a pro‐inflammatory state toward an immunoregulatory one. Furthermore, histological and molecular assessments revealed that the FMT was associated with enhanced mucosal barrier integrity. H&E staining showed that, unlike the severe epithelial erosion and inflammatory infiltration in the DSS group, the FMT group retained crypt architecture with lower histological damage scores (Figure 10p,l). The chemical barrier also recovered, with mucin‐secreting goblet cells preserved in AB‐PAS staining (Figure 10p). Neutrophil infiltration, a hallmark of acute colitis, was also notably reduced (Figure 10m,p). The physical barrier likewise improved: immunofluorescence showed that ZO‐1 and Occludin, disrupted and discontinuous in the model group, reorganized into a continuous, honeycomb‐like network with stronger fluorescence in FMT recipients (Figure 10n,o,q). Collectively, these findings provide functional evidence that the protective effects of ZnCA NPs are mediated, at least in part, by remodeling of the gut microbiota, which contributes to the suppression of inflammation and restoration of intestinal barrier integrity.

## 5 Conclusion

In summary, we developed a carrier‐free, coordination‐driven nanoplatform (ZnCA NPs) that integrates two dietary polyphenols, curcumin and anthocyanin, with Zn^2+^ for the management of ulcerative colitis. Coordination of Zn^2+^ as a multivalent node drove self‐assembly into a stable metal–phenolic network with a quasi‐amorphous structure, thereby improving the aqueous dispersibility and environmental stability of the constituent polyphenols. These particles combined efficient RONS scavenging with preferential accumulation in the inflamed colon, and in DSS‐induced colitis they conferred protection by simultaneously restoring the mucosal barrier and resolving the inflammatory microenvironment. Mechanistically, they suppressed the NF‐κB, MAPK, and JAK‐STAT pathways, lowered the pro‐inflammatory cytokines IL‐1β, IL‐6, and TNF‐α while raising IL‐10, and shifted the colonic transcriptome from a pro‐inflammatory toward a homeostatic state. They further preserved beneficial commensals and reversed DSS‐induced dysbiosis, and fecal microbiota transplantation provided functional evidence that this microbial remodeling contributes, at least in part, to the preventive benefit. Together with their favorable biocompatibility over the doses tested and their simple, carrier‐free fabrication, these properties position ZnCA NPs as a promising candidate for managing intestinal inflammation. Longer‐term studies of elemental pharmacokinetics and chronic accumulation will be needed to define the systemic safety margin, but this self‐assembled platform offers a versatile basis for future translational work in IBD and other oxidative‐stress‐related disorders.

## Author Contributions

X.H. and J.F. supervised the project and provided resources. Q.X., M.Y., and Y.C. performed the experiments. Q.X., J.W., X.L., and M.Y. analyzed the data. Q.X. wrote the manuscript with input from all authors. L.T. participated in the review and editing of the manuscript. All authors read and approved the final manuscript.

## Conflicts of Interest

The authors declare no competing interests.

## Use of Generative AI and AI‐Assisted Technologies in the Writing Process

The authors used Google Gemini 3.0 as a language‐editing aid to improve the clarity, grammar, and readability of the manuscript. This tool was not involved in the generation, analysis, or interpretation of any scientific data, nor in the formulation of research conclusions. The authors reviewed and edited the content and take full responsibility for the accuracy and integrity of the published article.

## Supporting information




**Supporting File**: advs77647‐sup‐0001‐SuppMat.docx.

## Data Availability

The raw 16S rRNA gene sequencing and transcriptomic datasets generated during this study have been deposited in the National Center for Biotechnology Information (NCBI) database. Microbiome sequencing data are accessible via the Sequence Read Archive (SRA) under BioProject accession number PRJNA1301881 (https://www.ncbi.nlm.nih.gov/bioproject/PRJNA1301881). The corresponding mouse colonic transcriptomic data have also been deposited in the SRA under BioProject accession number PRJNA1414944 (https://www.ncbi.nlm.nih.gov/bioproject/PRJNA1414944). Any other supporting data are available from the corresponding author upon reasonable request.
