# Supplementary material for: Supramolecular Zinc–Polyphenol Coordination Assemblies Mitigate Colonic Inflammation by Regulating Mucosal Barrier and Microbiome
Source: Adv Sci (Weinh). 2026 Sep 27:e77647. Online ahead of print. doi: 10.1002/advs.77647 (PMC13616285; doi:10.1002/advs.77647)
Supplement: Supplementary file 1 [file ADVS-9999-e77647-s001.docx]

**Supramolecular Zinc-Polyphenol Coordination Assemblies Mitigate Colonic Inflammation by Regulating Mucosal Barrier and Microbiome**

Qiwen Xie^1^, Mai Ye^1^, Xuexia Liu^2^, Jing Wang^1^, Ying Chen^1^, Jinheng Fu^1^*, Xiaochang Huang^1^**^,†,^***

^1^State Key Laboratory of Food Science and Resources, Nanchang University, Nanchang 330047, China.

^2^College of Life Sciences, Sichuan University, Chengdu, China.

**^†^** Lead contact: huangxiaochang@ncu.edu.cn

^*^Correspondence: fujinheng@ncu.edu.cn.

**Supporting experimental procedures**

**Evaluation of Aqueous Dispersibility**

To assess dispersion stability, excess free curcumin (Cur), curcumin-anthocyanin (CA) nanoparticles (NPs), and zinc-coordinated CA nanoparticles (ZnCA NPs) were each suspended in 5 mL of one of several media—deionized water, 1% (v/v) dimethyl sulfoxide (DMSO), and phosphate-buffered saline (PBS) at pH 2.0 and 7.4—by brief sonication. A 20-μL aliquot was withdrawn immediately (t = 0 h) as the baseline. After 2 h of undisturbed standing, a second 20-μL aliquot was withdrawn from the supernatant. Each aliquot was dissolved in 180 μL of methanol, and absorbance was measured at 430 nm. The dispersibility retention was quantified using the following equation:

Dispersibility retention = (A_2h_/A_0h_) × 100%.

**Thermal, UV Exposure, and pH Stability Studies**

To probe environmental stability, Cur, CA, and ZnCA dispersions were subjected to thermal, ultraviolet (UV), and alkaline stresses. Thermal treatment was performed in PBS (pH 7.4) in a water bath at 50 to 90 °C (10 °C increments) for 60 min. For photostability assays, samples sealed in transparent glass vials were placed under a 30 W UV light source for up to 24 h (sampling at 4, 8, 12, 16, and 24 h) with ambient light excluded. Additionally, pH sensitivity was examined by monitoring the formulations in 10 mM PBS (pH 8.0) over a 12-h period (0, 1, 4, 8, and 12 h). In all cases, residual Cur content was quantified from the optical density at 430 nm recorded on a UV-vis spectrophotometer [1].

**Quantitative Characterization of ZnCA Nanoparticles**

**Zinc Content Quantification by ICP-OES:** To determine the total zinc loading, we subjected 15.2 mg of lyophilized ZnCA nanoparticles to acid digestion. The digested sample was subsequently diluted to a final volume of 1.5 mL using deionized water. We quantified the zinc concentration using Inductively Coupled Plasma Optical Emission Spectrometry (ICP-OES).

**Cur Content Quantification by HPLC:** We quantified the retained curcumin content using a high-performance liquid chromatography system (HPLC, Thermo Fisher Ultimate 3000). Briefly, we dissolved 10.5 mg of ZnCA nanoparticles in 50 mL of methanol to ensure complete structural dissociation, followed by a 5-fold dilution with the mobile phase. The solution was filtered through a 0.22 μm membrane filter prior to injection. Chromatographic separation was achieved on a Symmetry C18 column (4.6 × 250 mm) maintained at 30 °C. The mobile phase consisted of acetonitrile and 0.1% phosphoric acid (50/50, v/v) delivered at a flow rate of 1.0 mL/min. The detection wavelength was set at UV 430 nm with an injection volume of 10 μL. Curcumin quantification was performed using a standard calibration curve spanning 5 to 100 ppm.

***In Vitro* Colloidal Stability in Simulated Gastrointestinal Fluids**

Colloidal stability of ZnCA NPs was assessed by measuring hydrodynamic diameter (Z‑average) and polydispersity index (PDI) after incubation in enzyme‑free simulated gastrointestinal fluids. Simulated gastric fluid (SGF, pH 1.2) and simulated intestinal fluid (SIF, pH 6.8) were prepared according to USP formulations but without enzymes, which would otherwise contribute background scattering in dynamic light scattering (DLS) measurements. The NP suspension was diluted into SGF or SIF and incubated at 37  °C with continuous shaking for 2 h. Size and PDI were then recorded by DLS at a scattering angle of 173° and a temperature of 25  °C. All measurements were performed in triplicate.

***In Vitro* Biomimetic Gastrointestinal Release Kinetics**

*In vitro* release of free Cur, CA NPs, and ZnCA NPs was studied by equilibrium dialysis. SGF (pH 1.2) and SIF (pH 6.8) containing 0.5% (w/v) Tween 20 were used as release media to maintain sink conditions. Two milliliters of each formulation (at a Cur concentration of 1 mg/mL) was placed in dialysis bags (MWCO 3500 Da), immersed in 20 mL of the respective medium, and shaken at 100 rpm and 37  °C (n = 3). At predetermined time intervals (0.5, 1, 2, 4, 6, 12, 24, 36, 48, and 72 h), 1 mL aliquots of the external release medium were collected, and an equivalent volume of fresh pre-warmed medium was immediately replenished to maintain a constant total volume. Cumulative release was monitored with a microplate reader (Thermo Scientific, USA) by measuring the optical density (OD) at the characteristic curcumin absorption wavelength (~430 nm). To correct for optical variation caused by co-pigmentation effects, the 100% cumulative-release benchmark for each group was defined as the total absorbance of the corresponding formulation fully disrupted and equilibrated under identical medium conditions. The cumulative release percentage (*Qn*) was calculated using the standard correction for the medium volume replaced during sequential sampling.

**Detailed Molecular Dynamics Simulation Protocol**

All molecular dynamics (MD) simulations were performed using GROMACS 2020.6, and the resulting trajectories were visualized and analyzed using Visual Molecular Dynamics (VMD) [1]. The initial configuration was generated using PACKMOL by randomly distributing six Zn^2+^ ions, acetate counterions, five curcumin (Cur) molecules, and fifteen cyanidin-3-O-glucoside (C3G) molecules in a cubic simulation box of 10 × 10 × 10 nm^3^ under periodic boundary conditions. C3G was selected as a representative anthocyanin molecule because the anthocyanin material used experimentally was a complex blueberry-derived extract. Counterions were included to neutralize the total charge of the system, which was subsequently solvated with explicit TIP3P water molecules. Partial atomic charges for the organic molecules were derived using the restrained electrostatic potential (RESP) method implemented in Multiwfn [2], and the corresponding molecular topology files were generated using ACPYPE. Zn^2+^ was represented using a non-bonded ion model, in which its interactions with the surrounding molecules were described by electrostatic and Lennard–Jones potentials. The system was initially subjected to energy minimization for 50,000 steps, followed by sequential equilibration under the canonical (NVT) and isothermal–isobaric (NPT) ensembles for 200 ps each. Long-range electrostatic interactions were treated using the particle mesh Ewald (PME) method, and van der Waals interactions were calculated using a cutoff of 1.0 nm. The temperature was maintained at 298 K using the velocity-rescaling thermostat, while the pressure during NPT equilibration was maintained at 1 bar using the Berendsen barostat. All bonds involving hydrogen atoms were constrained using the LINCS algorithm, and an integration time step of 2 fs was employed. Following equilibration, a 100 ns production simulation was conducted to investigate the aggregation behavior and intermolecular interactions among the ZnCA components. Solvent-accessible surface area (SASA), hydrogen bonding, electrostatic and van der Waals interaction energies, and representative structural configurations were analyzed from the resulting trajectories [1, 3, 4].

**Detailed Zebrafish Toxicity Assay Protocols**

The toxicity evaluation protocols were adapted from established methodologies with minor modifications [5-7]. Acute Toxicity Assay in Zebrafish Larvae: To evaluate acute systemic toxicity, zebrafish larvae at 3 days post-fertilization (dpf) were exposed to CA and ZnCA NPs. The NPs were dispersed in embryonic medium containing 0.1% (v/v) dimethyl sulfoxide (DMSO) and thoroughly homogenized to give a series of graded concentrations (50, 100, 300, 500, and 1000 μg/L). The exposure was performed in 6-well plates, where 30 larvae per well were immersed in 5 mL of the respective treatment solution, with each group analyzed in triplicate. A semi-static exposure regime was adopted, with the incubation medium renewed every 24 h. Larval survival rates were recorded over a 96-h exposure window. Embryonic Development Assay: Parallel experiments were conducted to evaluate the effects of NPs on embryonic development. Healthy zebrafish embryos at 2 hours post-fertilization (hpf) were transferred to 6-well plates and exposed to the identical concentration gradient of NPs (50, 100, 300, 500, and 1000 μg/L) prepared in the embryonic medium. Each well contained 30 embryos in 5 mL of solution, maintaining three independent replicates per treatment group. The exposure medium was refreshed daily, and the embryonic hatching rate was determined at 3 dpf. Across all experimental groups, the final DMSO vehicle concentration was held at 0.1% (v/v) to avoid confounding vehicle-induced toxicity.

**Detailed Histological and Histochemical Staining Protocol**

To evaluate colonic mucosal architecture and goblet cell abundance, histological and histochemical analyses were performed on routine paraffin sections. Immediately post-euthanasia, murine colon segments were excised, cleared of luminal contents by gentle flushing with ice-cold PBS, and fixed in 4% paraformaldehyde at 4 °C for 24 h. Fixed specimens were subsequently dehydrated through an ascending ethanol gradient (75%, 85%, 95%, and 100% v/v), cleared in two changes of xylene, and embedded in paraffin blocks. Individual blocks were sectioned at 4 µm using a microtome. Prior to staining, the 4-µm sections were deparaffinized and rehydrated through a descending ethanol series. General tissue morphology and inflammatory cell infiltration were evaluated by hematoxylin and eosin (H&E) staining (G1100, Solarbio, Beijing, China). Colonic goblet cells and mucus production were visualized using an Alcian Blue-Periodic Acid Schiff (AB-PAS) histochemical staining kit (G1285, Solarbio) in strict accordance with the manufacturer's instructions. Stained slides were digitized with a NanoZoomer S60 digital pathology scanner (Hamamatsu Photonics, Hamamatsu, Japan) to capture high-resolution images. Histological scoring of colitis severity was conducted by an investigator blinded to the experimental groupings, according to the grading criteria detailed in Table S3 [3].

**Detailed Immunofluorescence Staining Protocol**

Murine colon tissue sections (4 μm) were deparaffinized in xylene and rehydrated through graded ethanol for the assessment of tight junction integrity. Antigen retrieval was achieved by heating at 95 °C for 15 min in citrate buffer (pH 6.0). Following a 10-min permeabilization step using 0.2% Triton X-100, non-specific epitopes were blocked with 5% normal goat serum for 1 h at ambient temperature. The sections were then incubated with rabbit anti-occludin (1:200) and rabbit anti-ZO-1 (1:150) primary antibodies overnight at 4 °C. Detection was performed by incubating the sections with an Alexa Fluor 488-conjugated goat anti-rabbit IgG secondary antibody (1:500) for 1 h in the dark. Slides were then stained with DAPI for nuclear visualization and mounted with anti-fade mounting medium. Whole-slide fluorescence imaging was conducted using a PANNORAMIC MIDI/SCAN 150 digital slide scanner (3DHISTECH, Hungary).

**Detailed Immunohistochemistry Protocol**

To assess inflammatory cell infiltration within the murine intestinal barrier, immunohistochemistry (IHC) was performed for myeloperoxidase (MPO). Paraffin-embedded colonic blocks were sliced into 4-μm sections, which were subsequently deparaffinized in xylene and rehydrated through graded ethanol. Heat-induced epitope retrieval was achieved in a microwave oven using a pH 6.0 citrate solution (8 min at medium power and 7 min at medium-low power). To eliminate background interference, endogenous peroxidase activity was quenched with 3% H2O2 for 25 min in the dark. Following a standard PBS rinse, the sections were blocked with 3% BSA for 30 min and then incubated overnight at 4 °C with a primary antibody against MPO. Unbound primary antibodies were removed by washing with PBS three times, and the bound antibodies were detected with an HRP-conjugated secondary antibody for 50 min at room temperature. Proliferation and infiltration patterns were visualized by DAB chromogenic development (brown). Lastly, cell nuclei were stained with hematoxylin for ~3 min, and the slides were coverslipped with neutral resin after ascending dehydration and xylene clearing. All histological imaging was performed using a PANNORAMIC MIDI/SCAN 150 digital slide scanner (3DHISTECH, Hungary).

**Detailed RNA-seq Library Construction and Bioinformatics Pipeline**

Total RNA was extracted from murine colonic specimens using TRIzol Reagent (Invitrogen, Carlsbad, CA, USA), followed by an immediate digestion step with DNase I (Takara, Dalian, China) to eliminate residual genomic DNA. RNA concentration and purity were assessed using a NanoDrop spectrophotometer (Thermo Fisher Scientific, Waltham, MA, USA). Subsequently, RNA integrity was further assessed by microfluidic capillary electrophoresis on an Agilent 2100 Bioanalyzer using the RNA Nano 6000 Assay Kit (Agilent Technologies, Santa Clara, CA, USA). Only RNA samples with an RNA Integrity Number (RIN) ≥ 8.0, alongside an OD260/280 ratio of 1.8–2.2, and an OD260/230 ratio ≥ 2.0, were used for downstream library construction. Transcriptome libraries were constructed from 1 μg of total RNA per sample by Novogene Co., Ltd. (Beijing, China) using the TruSeq Stranded mRNA Library Prep Kit (Illumina, San Diego, CA, USA) according to the manufacturer’s instructions. Briefly, polyadenylated mRNA transcripts were selectively captured using oligo(dT) magnetic beads and subjected to thermal fragmentation. First-strand cDNA was primed with random hexamers, followed by second-strand synthesis to generate double-stranded cDNA (dscDNA). The resulting dscDNA fragments underwent end-repair, 3′ A-tailing, and ligation with Illumina-compatible indexing adapters. Ligated fragments of approximately 300 bp were size-selected by 2% agarose gel electrophoresis. Libraries were amplified by 15 cycles of PCR using Phusion High-Fidelity DNA Polymerase (New England Biolabs, Ipswich, MA, USA), with final library profiles validated on the Agilent 2100 Bioanalyzer. Multiplexed sequencing was performed on an Illumina NovaSeq 6000 platform in a 150 bp paired-end (PE150) configuration, yielding a minimum raw sequencing depth of 40 million reads per sample [5].

**RNA-seq analysis**

To ensure reliable downstream analysis, raw sequence reads were subjected to stringent quality filtering. Adapters were removed using SeqPrep (v1.1), and low-quality regions (Phred score < 20) were trimmed using Sickle (v1.33), with reads (< 50 bp) discarded. The resulting high-quality clean reads were mapped to the *Mus musculus* reference genome (GRCm39) using HISAT2 (v2.2.1) in strand-specific mode. Transcript abundance was quantified using RSEM (v1.3.3) as Transcripts Per Million (TPM) values. For differential expression profiling, the raw expected counts from RSEM were imported into the DESeq2 package (v1.34.0) in the R environment. Differentially expressed genes (DEGs) between designated experimental groups were screened based on the statistical thresholds of an absolute log_2_-transformed fold change (|log_2_FC|) ≥ 1 and adjusted p-value < 0.05. To interpret the biological functions associated with these DEGs, Gene Ontology (GO) and Kyoto Encyclopedia of Genes and Genomes (KEGG) pathway enrichment analyses were performed using Goatools (v1.2.3) and KOBAS (v3.0), respectively. Enriched terms were considered significant at a Bonferroni-corrected p-value threshold of < 0.05 [3].

**Detailed 16S rRNA Amplicon Sequencing and Analysis**

For community profiling, fresh murine fecal samples were collected in sterile cryovials, flash-frozen in liquid nitrogen, and stored at -80 °C until processing. Total microbial genomic DNA was extracted from 200 mg of each fecal sample by Novogene Co., Ltd. (Beijing, China) using the E.Z.N.A.® Soil DNA Kit (Omega Bio-tek). DNA concentration and purity were assessed on a NanoDrop 2000 spectrophotometer (Thermo Fisher Scientific, Waltham, MA, USA) to ensure A_260_/A_280_ and A_260_/A_230_ ratios fell within 1.8–2.0 and ≥ 1.7, respectively, while DNA integrity was verified by 1% agarose gel electrophoresis. Amplification of the bacterial 16S rRNA V3-V4 hypervariable regions was performed using 338F (5'-ACTCCTACGGGAGGCAGCAG-3') and 806R (5'-GGACTACHVGGGTWTCTAAT-3') primers. Triplicate 20-μL PCR mixtures—comprising 10 ng of template, 4 μL of 5× FastPfu Buffer, 2 μL of dNTPs (2.5 mM), 0.8 μL of each primer (5 μM), and 0.4 μL of FastPfu High-Fidelity Polymerase (TransGen Biotech)—were subjected to a cycling regime of 95 °C for 3 min, 27 cycles of 95 °C for 30 s, 55 °C for 30 s, 72 °C for 45 s, and a 10-min final extension at 72 °C. Pooled amplicons were purified using the AxyPrep DNA Gel Extraction Kit (Axygen) and quantified using a QuantiFluor™-ST fluorometer (Promega). Barcoded sequencing libraries were subsequently prepared by adapter ligation, pooled equimolarly, and subjected to paired-end sequencing on an Illumina NovaSeq 6000 platform, generating at least 100,000 raw read pairs per sample. Raw reads were demultiplexed into individual sample files according to their index sequences for downstream bioinformatic analysis.

Microbiome amplicon data were processed using the QIIME 2 pipeline (version 2024.2) [8]. Following quality filtering, the q2-deblur plugin was used for sequence denoising and the generation of amplicon sequence variants (ASVs) [9]. Taxonomic assignment of representative ASV sequences was performed using a pre-trained Naïve Bayes classifier against the Greengenes2 database (version 2024.09) [10]. Differentially abundant taxa across groups were identified using ANCOM-BC (version 2.8.1) [11]. Spearman’s rank correlation coefficients were calculated to assess potential associations between the relative abundances of key bacterial taxa and host clinical parameters, including colonic inflammatory markers.

**Biodistribution and Targeting Studies**

To track the gastrointestinal transit and evaluate the colon-targeting efficiency of the nanoplatforms, the hydrophobic near-infrared (NIR) fluorophore, 1,1'-dioctadecyl-3,3,3',3'-tetramethylindotricarbocyanine iodide (DiR), was physically encapsulated within the self-assembled CA and ZnCA matrices to yield optically trackable nanoprobes. This lipophilic dye-loading strategy enabled non-invasive, longitudinal fluorescence imaging of the relative fluorescence distribution and gastrointestinal retention of the formulations in mice.

**Preparation of DiR-loaded Nanoparticles:** To fabricate optically trackable nanoprobes for biodistribution tracking, DiR-loaded CA (DiR@CA) and ZnCA (DiR@ZnCA) nanoparticles were synthesized by incorporating the near-infrared lipophilic dye into the multi-component self-assembly process. Typically, curcumin (368.4 mg) and anthocyanin (861.8 mg) were co-dissolved with DiR in a DMSO/ultrapure water vehicle (3:2, v/v) at a theoretical tracer concentration of 500 μg/mL. For the coordinated DiR@ZnCA networks, zinc acetate dihydrate (21.95 mg) was added to the initial feedstock, whereas this precursor was omitted to yield the uncoordinated DiR@CA control. Driven by a combination of hydrophobic interactions, π-π stacking, and metal–phenolic coordination, the reaction mixtures were incubated at 70 °C for 10 min under vigorous magnetic stirring, followed by 5 min of ultrasonication to promote supramolecular assembly. To remove the organic co-solvent, unreacted monomers, and non-encapsulated fluorophore, the resulting colloidal solutions were dialyzed against excess ultrapure water for 48 h in a light-shielded environment using a 3500 Da molecular weight cut-off (MWCO) membrane. The solid DiR@CA and DiR@ZnCA nanoparticles were finally recovered by lyophilization.

***In Vivo* Imaging:** To assess the gastrointestinal transit and colon-targeting specificity of the nanoplatforms, male C57BL/6 mice (6–8 weeks old, 20–22 g) were used in accordance with protocols approved by the Institutional Animal Care and Use Committee. The study evaluated both healthy animals and mice with dextran sulfate sodium (DSS)-induced colitis. Animals were randomized into four groups (n = 9 per group): (1) healthy controls administered DiR@ZnCA; (2) colitis models administered free DiR; (3) colitis models administered DiR@CA; and (4) colitis models administered DiR@ZnCA. Before imaging, all mice were fasted overnight with *ad libitum* access to water to minimize background gastrointestinal autofluorescence from regular chow. The respective formulations—comprising free DiR dissolved in a 1% (v/v) DMSO/aqueous vehicle and the self-assembled nanoparticle dispersions—were administered by intragastric gavage at an equivalent DiR dose of 2 mg/kg body weight. At predetermined post-administration intervals (3, 6, 12, and 24 h), the mice were anesthetized by isoflurane inhalation. Non-invasive fluorescence images were acquired using an IVIS Spectrum imaging system (PerkinElmer, Waltham, MA, USA) with excitation and emission wavelengths of 760 and 790 nm, respectively.

***Ex Vivo* Imaging:** For high-resolution mapping of gastrointestinal transit, mice were sacrificed at scheduled post-gavage intervals (3, 12, and 24 h; n = 3 mice per treatment group at each time point). The intact gastrointestinal tract (stomach, small intestine, and colon) was promptly excised and imaged *ex vivo* under the same filter settings used for *in vivo* imaging. Colonic accumulation and targeting efficiency of the nanoplatforms were quantified by drawing regions of interest (ROIs) over the isolated colon segments. Relative fluorescence intensities in the isolated colon segments were semi-quantified using Image-Pro Plus 6.0 software (Media Cybernetics, USA) by defining consistent regions of interest (ROIs), with identical analysis parameters applied to all images.

**Detailed Protocol for Fecal Microbiota Transplantation**

To assess whether the protective effects of high-dose ZnCA nanoparticles were transferable through the gut microbiota, a fecal microbiota transplantation (FMT) experiment was conducted [9]. Donor fecal material was collected from the ZnCA (H) group beginning three days after treatment cessation to minimize potential interference from residual nanoparticles. Fresh fecal pellets were homogenized in sterile PBS, filtered through a 70 μm strainer, and centrifuged to isolate the bacterial fraction. The resulting pellet was washed and resuspended to a final concentration of 20 mg/mL to serve as the transplant material. Antibiotic-treated, microbiota-depleted recipient mice were established by administering a broad-spectrum antibiotic cocktail containing ampicillin (1 g/L), neomycin (1 g/L), metronidazole (1 g/L), and vancomycin (0.5 g/L) in the drinking water for three weeks, followed by a two-day washout period.

Subsequently, the microbiota-depleted recipients were randomly assigned to three groups: a negative control (NC) group, a DSS model group, and the FMT-treated (DSS+FMT-NP) group. Colitis was induced in the latter two groups by *ad libitum* access to 3.5% (*w/v*) DSS solution, while the NC group received normal drinking water. Concurrently, the DSS+FMT-NP group received a daily oral gavage of 200 μL of the prepared fecal suspension, whereas the NC and DSS groups received an equivalent volume of sterile PBS. Clinical parameters, including body weight and disease activity index (DAI), were monitored daily. At the experimental endpoint, mice were euthanized to harvest colon tissues for macroscopic (length measurement), histological (H&E staining), and biochemical analyses. Mucosal barrier integrity was evaluated by immunofluorescence staining of tight junction proteins (ZO-1 and OCCLUDIN), while inflammation was characterized by quantifying MPO activity by immunohistochemistry and cytokine levels (TNF-α, IL-1β, IL-6, and IL-10) using ELISA. Additionally, 16S rRNA gene sequencing of cecal contents was performed to confirm colonization by the transplanted microbiota.

**Table supplementary information**

**Table S1.** RT-qPCR primer sequences

| Primer | Sequence (5′-3′) | Reference |
| --- | --- | --- |
| *tnf-α* | F: TCTGAACTGACTGAGGAACAAG  R: AAGTGCTGTGGTCGTGTCT | [13] |
| *il-10* | F: GGAGACCATTCTGCCAACAG  R: CACCATATCCCGCTTGAGTTC | [14] |
| *il-6* | F: GTCTGCTACACTGGCTACACT  R: CACATCCTGAACTTCGTCTCC | [13] |
| *il-1β* | F: CTCCGCTCCACATCTCGTA  R: TCCTGTCCATCTCCACCATC | [13] |
| β-actin | F: TGGCATCACACCTTCTACAATG |  |
|  | R: CACCAGAGTCCATCACAATACC |  |

**Table S2.** Disease activity index(DAI) scoring

| Score | Body weight loss (a) | Stool consistency (b) | Visible blood in stool (c) |
| --- | --- | --- | --- |
| 0 | None | Normal | No visible blood |
| 1 | 1%-5% | Soft and moist, but well-formed stools | Trace amounts of blood on the fecal surface |
| 2 | 6%-10% | Very soft and partially formed stools | Mild but clearly visible blood in the stool |
| 3 | 11%-15% | Unformed, paste-like stools | Moderate blood distributed within or around the stool |
| 4 | >15% | Watery diarrhea | Severe gross bleeding or rectal bleeding |
| DAI= (a + b + c) /3 | | | |

**Table S3.** H&E scoring

| Score | Epithelial Injury (a) | Loss of crypt architecture (b) | Inflammatory Infiltration (c) |
| --- | --- | --- | --- |
| 0 | None | Normal | Absent |
| 1 | Mild | Mild reduction | Focal |
| 2 | Moderate | Marked reduction | Moderate |
| 3 | Severe | Near-complete loss | Extensive |
| Total histological score = a + b + c (range: 0–9) | | | |

**Table S4.** Measured and estimated composition of the optimized ZnCA nanoparticles.

| Component | Content (mg/g) | Mass fraction (wt.%) | Encapsulation Efficiency (%) |
| --- | --- | --- | --- |
| Zinc (Zn^2+^) | 15.36 | 1.54 | — |
| Cur | 506.70 | 50.67 | 59.7% |
| Ant^a^ | 402.50^a^ | 40.25^a^ | — |
| TGA-derived mass loss ^b^ | 75.40 | 7.54 | — |
| Total | — | 100.00 | — |

Notes: ^a^ Estimated by mass balance after subtracting the measured Zn and Cur contents and the TGA-derived mass loss below 250 °C; this value represents the anthocyanin-rich extract-associated fraction rather than pure anthocyanins.

^b^ TGA-derived mass loss below 250 °C may include moisture, residual volatiles, and partial decomposition of organic components. Zn and Cur contents were determined by ICP–OES and HPLC, respectively. Cur encapsulation efficiency was calculated relative to the initial feeding amount.

**Table S5.** ^1^H-NMR Chemical Shifts and Perturbations of Key Protons in Different Assembly States

| Proton Assignment | Free Cur (δ) | ZnCur (δ/Δδ) | CA (δ/Δδ) | ZnCA (δ/Δδ) |
| --- | --- | --- | --- | --- |
| H-13, 7 (β-vinyl) | 7.56, 7.52 | 7.42, 7.40 / -0.14, -0.12 | 7.56, 7.53 / -0.00, +0.01 | 7.43, 7.41 / -0.13, -0.11 |
| H-19, 2 (Ar) | 7.32 | 7.06 / -0.26 | 7.32 / -0.00 | 7.05 / -0.27 |
| H-6, 15 (Ar) | 7.15 | 6.95 / -0.20 | 7.15 / -0.00 | 6.94 / -0.21 |
| H-18, 3 (Ar) | 6.83, 6.81 | 6.67, 6.65 / -0.16 | 6.83, 6.81 / -0.00, 0.00 | 6.66 / -0.17, -0.15 |
| H-12, 8 (α-vinyl) | 6.77, 6.73 | 6.48, 6.46 / -0.29, -0.27 | 6.77, 6.73 / -0.00 | 6.48, 6.45 / -0.29, -0.28 |
| H-10 (Enol-CH) | 6.06 | 5.81 / -0.25 | 6.06 / -0.00 | 5.81 / -0.25 |
| -OCH_3_ (Methoxy) | 3.83, 3.32 | 3.75, 3.15 / -0.08, -0.17 | 3.83, 3.32 / -0.00 | 3.75, 3.15 / -0.08, -0.17 |

**References**

[1]	Y. Hu, Y. Miao, Y. Zhang, X. Wang, X. Liu, W. Zhang, D. Deng, *ACS Nano* **2024**, *18* (40), 27557, https://doi.org/10.1021/acsnano.4c08508.

[2]	J. Yang, S. Tan, S. Ge, M. Yang, H. Liu, W. Liu, K. Zhang, Z. Zhang, Z. H. Wang, J. Shi, J. Liu, *Proc Natl Acad Sci U S A* **2024**, *121* (52), e2403417121, https://doi.org/10.1073/pnas.2403417121.

[3] S. Gao, H. Zheng, S. Xu, J. Kong, F. Gao, Z. Wang, Y. Li, Z. Dai, X. Jiang, X. Ding, H. Lei, *Adv Healthc Mater* **2023**, *12* (31), e2301826, https://doi.org/10.1002/adhm.202301826.

[4] Y. Yang, M. Yu, Y. Mo, Y. Cheng, B. Huang, W. Wang, M. Zhu, X. Jia, L. Feng, B. Yang, *Carbohydr Polym* **2024**, *346*, 122658, https://doi.org/10.1016/j.carbpol.2024.122658.

[5] H. Chen, P. Lei, H. Ji, J. Ma, Y. Fang, H. Yu, J. Du, L. Qu, Q. Yang, L. Luo, K. Zhang, W. Wu, L. Jin, D. Sun, *Life Sci* **2023**, *329*, 121956, https://doi.org/10.1016/j.lfs.2023.121956.

[6] M. A. Eissa, Y. Z. H. Hashim, M. H. Mohd Nasir, Y. A. Nor, H. M. Salleh, M. L. M. Isa, S. S. S. Abd-Azziz, N. M. Abd Warif, E. Ramadan, N. M. Badawi, *Drug Deliv* **2021**, *28* (1), 2618, https://doi.org/10.1080/10717544.2021.2012307.

[7] Y. Tang, Y. Zhan, S. Gao, T. Li, H. Xuan, *J Hazard Mater* **2025**, *494*, 138695, https://doi.org/10.1016/j.jhazmat.2025.138695.

[8]	E. Bolyen, J. R. Rideout, M. R. Dillon, N. A. Bokulich, C. C. Abnet, G. A. Al-Ghalith, H. Alexander, E. J. Alm, M. Arumugam, F. Asnicar, Y. Bai, J. E. Bisanz, K. Bittinger, A. Brejnrod, C. J. Brislawn, C. T. Brown, B. J. Callahan, A. M. Caraballo-Rodríguez, J. Chase, E. K. Cope, R. Da Silva, C. Diener, P. C. Dorrestein, G. M. Douglas, D. M. Durall, C. Duvallet, C. F. Edwardson, M. Ernst, M. Estaki, J. Fouquier, J. M. Gauglitz, S. M. Gibbons, D. L. Gibson, A. Gonzalez, K. Gorlick, J. Guo, B. Hillmann, S. Holmes, H. Holste, C. Huttenhower, G. A. Huttley, S. Janssen, A. K. Jarmusch, L. Jiang, B. D. Kaehler, K. B. Kang, C. R. Keefe, P. Keim, S. T. Kelley, D. Knights, I. Koester, T. Kosciolek, J. Kreps, M. G. I. Langille, J. Lee, R. Ley, Y. X. Liu, E. Loftfield, C. Lozupone, M. Maher, C. Marotz, B. D. Martin, D. McDonald, L. J. McIver, A. V. Melnik, J. L. Metcalf, S. C. Morgan, J. T. Morton, A. T. Naimey, J. A. Navas-Molina, L. F. Nothias, S. B. Orchanian, T. Pearson, S. L. Peoples, D. Petras, M. L. Preuss, E. Pruesse, L. B. Rasmussen, A. Rivers, M. S. Robeson, 2nd, P. Rosenthal, N. Segata, M. Shaffer, A. Shiffer, R. Sinha, S. J. Song, J. R. Spear, A. D. Swafford, L. R. Thompson, P. J. Torres, P. Trinh, A. Tripathi, P. J. Turnbaugh, S. Ul-Hasan, J. J. J. van der Hooft, F. Vargas, Y. Vázquez-Baeza, E. Vogtmann, M. von Hippel, W. Walters, Y. Wan, M. Wang, J. Warren, K. C. Weber, C. H. D. Williamson, A. D. Willis, Z. Z. Xu, J. R. Zaneveld, Y. Zhang, Q. Zhu, R. Knight, J. G. Caporaso, *Nat Biotechnol* **2019**, *37* (8), 852, https://doi.org/10.1038/s41587-019-0209-9.

[9]	B. J. Callahan, P. J. McMurdie, M. J. Rosen, A. W. Han, A. J. Johnson, S. P. Holmes, *Nat Methods* **2016**, *13* (7), 581, https://doi.org/10.1038/nmeth.3869.

[10]	C. Quast, E. Pruesse, P. Yilmaz, J. Gerken, T. Schweer, P. Yarza, J. Peplies, F. O. Glöckner, *Nucleic Acids Res* **2013**, *41* (Database issue), D590-6, https://doi.org/10.1093/nar/gks1219.

[11]	N. Segata, J. Izard, L. Waldron, D. Gevers, L. Miropolsky, W. S. Garrett, C. Huttenhower, *Genome Biol* **2011**, *12* (6), R60, https://doi.org/10.1186/gb-2011-12-6-r60.

[12]	S. Zhong, Y. Q. Sun, J. X. Huo, W. Y. Xu, Y. N. Yang, J. B. Yang, W. J. Wu, Y. X. Liu, C. M. Wu, Y. G. Li, *Imeta* **2024**, *3* (2), e180, https://doi.org/10.1002/imt2.180.

[13]	S. P. R. R. Nayak, L. S. Dhivya, R. R, B. O. Almutairi, S. Arokiyaraj, M. K. Kathiravan, J. Arockiaraj, *European Journal of Pharmacology* **2023**, *957*, https://doi.org/10.1016/j.ejphar.2023.175994.

[14]	S. Brugman, K. Y. Liu, D. Lindenbergh-Kortleve, J. N. Samsom, G. T. Furuta, S. A. Renshaw, R. Willemsen, E. E. Nieuwenhuis, *Gastroenterology* **2009**, *137* (5), 1757, https://doi.org/10.1053/j.gastro.2009.07.069.

**Figure S1. Stability and *in vitro* release profiles of free Cur, CA NPs, and ZnCA NPs under diverse simulated environmental conditions.** (a) Thermal stability of formulations in PBS (pH 7.4) after incubation at temperatures ranging from 50 to 90 °C for 60 min. (b) UV photostability assessment of samples exposed to a 30 W UV lamp for up to 24 h. (c) pH stability profiles in an alkaline environment (10 mM PBS, pH 8.0) over a 12-h incubation period. (d) Particle size distribution profiles (including Z-average diameter and PDI) of ZnCA NPs after a 2-h incubation in enzyme-free simulated gastric fluid (SGF, pH 1.2) and simulated intestinal fluid (SIF, pH 6.8) at 37 °C. (e) *In vitro* biomimetic gastrointestinal release kinetics of free Cur, CA NPs, and ZnCA NPs in SGF and SIF containing 0.5% (w/v) Tween 20 over a 72-h period. Data in (a–c, e) are presented as mean ± SD (n = 3).

**Figure S2. Relative organ weights of mice in different treatment groups.** Statistical analysis of the relative weights of (a) heart, (b) liver, (c) kidney, and (d) lung. Data are presented as mean ± SD (n = 4-5). ns indicates no significant difference among all groups.

**Figure S3. Gene Set Enrichment Analysis (GSEA) of inflammatory signaling pathways.** Enrichment plots for (a) NF-κB, (b) MAPK, (c) JAK-STAT, and (d) TNF signaling pathways. The analysis compares the gene expression profiles between the DSS group and the Control group (DSS vs. Con). NES and *P_adj_* denote the normalized enrichment score and adjusted *P*-value, respectively.

**Figure S1**

**Figure S2**

**Figure S3**
